# Exploring Feed Efficiency in Beef Cattle: From Data Collection to Genetic and Nutritional Modeling

**DOI:** 10.3390/ani14243633

**Published:** 2024-12-17

**Authors:** Ayooluwa O. Ojo, Henrique A. Mulim, Gabriel S. Campos, Vinícius Silva Junqueira, Ronald P. Lemenager, Jon Patrick Schoonmaker, Hinayah Rojas Oliveira

**Affiliations:** 1 Department of Animal Sciences, Purdue University, West Lafayette, IN 47907, USA; ojo0@purdue.edu (A.O.O.); hmulim@purdue.edu (H.A.M.); gabrielsoarescampos@hotmail.com (G.S.C.); rpl@purdue.edu (R.P.L.); jschoonm@purdue.edu (J.P.S.); 2 Department of Animal Biosciences, Interbull Centre, 75007 Uppsala, Uppland, Sweden; 3 R&D Department, Bayer Crop Science, Uberlândia 38400-299, Minas Gerais, Brazil; viniciussilva.junqueira@bayer.com

**Keywords:** feed intake, genomics, precision livestock farming, resource management, sustainability

## Abstract

Increasing feed efficiency in beef cattle is critical for meeting the growing global demand for beef while managing rising feed costs and environmental impacts. Challenges in recording feed intake and combining genomic and nutritional models hinder improvements in feed efficiency for sustainable beef production. This review examines the progression from traditional data collection methods to modern genetic and nutritional approaches that enhance feed efficiency. We first discuss the technological advancements that allow precise measurement of individual feed intake and efficiency, providing valuable insights for research and industry. The role of genomic selection in identifying and breeding feed-efficient animals is then explored, emphasizing the benefits of combining data from multiple populations to enhance genomic prediction accuracy. Additionally, the paper highlights the importance of nutritional models that could be used synergistically with genomic selection. Together, these tools allow for optimized feed management in diverse production systems. Combining these approaches also provides a roadmap for reducing input costs and promoting a more sustainable beef industry.

## Simple Summary

As global demand for beef rises, cattle farmers face growing pressure to reduce costs, manage resources wisely, and maintain environmentally friendly practices. Feed efficiency, or the ability of cattle to gain weight while consuming less feed, has become a key solution to these challenges, as feed represents a major production cost. However, difficulties in measuring feed intake and addressing maintenance, reproduction, and productivity limit feed efficiency improvements for sustainable beef production. This paper reviews advancements in measuring and improving feed efficiency in beef cattle, from technologies that track how much individual animals eat to breeding methods that identify cattle with better genetic potential for feed efficiency. Using tools such as genomic data, scientists and producers can now predict which cattle will grow well on less feed, reducing the resources needed and lowering costs. Nutritional models are also helping producers optimize feeding strategies based on cattle needs, improving both efficiency and animal health. Combining these approaches, this review paper offers a roadmap for a more profitable and sustainable beef industry that can meet future demands without exhausting natural resources, and therefore benefiting producers and consumers.

## 1. Introduction

The global beef (meat) supply decreased by 11.25% between 2010 and 2015 [1], then had a 19.2% increase from 2015 to 2022 [1]. In 2024, the United States produced 20% of the world’s beef supply, with only Brazil, China, and the European Union each producing more than 10% [2]. By 2030, the global population is projected to exceed 8.5 billion, reaching over 9.8 billion by 2050 [3], with worldwide beef consumption estimated to be between 460 and 570 million tons [4,5]. Considering the global population has already surpassed 7 billion, beef (meat) consumption would be twice as high as in 2008 [4,5], despite urban sprawl reducing the total area of farmland available for agricultural production compared to the 1970s [6,7]. Unfortunately, the United States and global beef producers face numerous challenges, including climate change, rising energy costs, non-agricultural encroachment on crop and grazing land, greater competition for feed sources, and confusing governmental policies [8]. These challenges significantly impact the prices of feed, which account for up to 75% of the total cost of producing beef cattle [9]. Cattle play a crucial role in utilizing the vast grazing land by harvesting grasses and producing a nutritious protein source while preserving the land for future generations. It is, therefore, essential to recognize the diverse challenges cattle producers face as they strive to maintain a sustainable business while upholding animal welfare, ensuring a safe and healthy beef supply, and conserving the environment. Additionally, it emphasizes the importance of continuous genetic progress of economically important traits that enable cattle producers to achieve greater efficiency with fewer resources [9].

Over the past four decades, there has been an increasing focus on feed efficiency (FE) among scientists and the industry. For producers, enhancing FE represents a shift in mindset, moving from solely considering revenue generation to actively managing costs. Improving FE not only boosts profitability but also helps to reduce the substantial expenses associated with feed [10]. Specifically in beef cattle, improved FE reduces costs and enhances the production system’s overall efficiency [11]. Studies have demonstrated that modest improvements in FE can have a substantial economic impact on beef production [11]. A 5% increase in FE can have four times the economic impact of a 5% increase in average daily weight gain (ADG) [12,13]. Similarly, research performed in feedlot settings revealed that a 10% increase in ADG led to an 18% increase in profitability [14]. In comparison, a 10% increase in FE led to a substantial 43% increase in profits [14]. These results highlight the significance of improving the efficiency of feed and forage utilization in cattle production to reduce input costs.

The nutritional requirements of beef cattle, such as energy, protein, and minerals, change depending on their growth stage. This variation occurs because the proportions and composition of the animal’s body evolve over time, with different tissues and organs growing at different rates. As a result, young animals have distinct nutritional needs compared to adults, both in terms of the types of nutrients required and the quantities of each component. Approximately 70 to 75% of the nutrients consumed by beef cattle are allocated to body maintenance functions [15,16]. Historically, studies on FE have primarily focused on young, growing, and finishing cattle, neglecting the importance of the cow herd and its maintenance requirements. As a cow goes through different stages of its life, such as growing, breeding, gestation, and lactation, its nutritional requirement changes accordingly [17]. Therefore, to improve the profitability of the beef cattle industry, it is crucial to consider these changes in the overall FE of the production system [18]. Furthermore, accurately estimating the nutritional requirements for modern genotypes under current feeding conditions is of utmost importance in enhancing the profitability of the beef industry [17]. Measuring individual animal feed intake (FI) has traditionally been challenging and costly. However, recent advancements in computing and electronics and the availability of reliable automatic FI recorders have simplified the process [18].

The livestock industry has significantly increased access to genomic data, creating a profitable method for estimating the genetic merit of young animals early in life. This is beneficial for making selection decisions that promote genetic gains. Genomic selection has become widespread in animal breeding programs because it facilitates the selection of traits that are complex and expensive to measure, such as FE [19]. Genomic selection reduces the generation interval and consequently increases genetic gains by allowing breeders to predict the genetic potential of animals early in their lifetimes [19,20]. In beef and dairy cattle, the accuracy of genomic predictions for residual feed intake (RFI) and dry matter intake (DMI) was reported to range from 0.2 to 0.4 [21,22,23,24,25,26,27,28,29]. RFI’s phenotypic independence from daily gain and heritability estimates of 0.08 to 0.49 among cattle populations make it a preferred measure for dissecting FE biology and genomic selection. There are opportunities to enhance the precision of these forecasts by utilizing data from multiple research populations. Using data from nine *Bos taurus taurus* and *Bos taurus indicus* cattle breeds, Bolormaa et al. [23] estimated a 0.36 genomic prediction accuracy for RFI. It has been demonstrated that combining data from three research herds in Australia and Europe improves the accuracy of genomic prediction for DMI. It increased from 0.33 when utilizing data from a single country to 0.35 from all three countries [27,30]. Despite significant advancements in the study and application of FE in beef cattle, combining genomic data and nutritional modeling still faces challenges in terms of cost and practical implementation. Furthermore, the complex interaction between maintenance requirements, reproductive efficiency, and lifetime productivity in mature animals remains underexplored. Addressing these gaps is critical to fully leveraging FE improvements for economic and environmental sustainability in beef production. Therefore, this review paper aims to summarize advancements in measuring and improving FE in beef cattle. It explores innovations in data collection, the role of genomic selection in breeding more feed-efficient animals, and the integration of nutritional models to optimize feed use under various conditions. By combining these approaches, the paper highlights strategies for enhancing profitability and sustainability in response to increasing global demand and resource constraints in beef production.

## 2. Measures of Feed Efficiency

FI and production outputs are correlated, and examining them in isolation provides limited insight into production system efficiency. Due to the variation in the FI of individual animals, knowledge of body weight (BW) and production level alone is not enough to estimate FI in national cattle evaluation systems [31]. Thus, there is a need for accurate measurement of FI. Researchers often focus on specific production cycle phases to compare FI and production outputs, using an index that combines these factors to express FE. Multiple definitions of FE have been proposed in both literature and industry. As such, the term “*feed efficiency*” seems vague and needs to be distinguished between the proposed indices. When comparing the proposed alternative definitions, the key distinction lies in whether they involve measuring actual FI or not [9]. Extensive research indicates that the most useful definitions of efficiency require accurate measurement of individual FI [9]. For instance, dry matter measurements are needed alongside nutrient intake to calculate FE. Berry and Crowley [32] comprehensively reviewed different methods for calculating FE and classified them into ratios and residual or regression traits. One example of a ratio trait is the feed conversion ratio (FCR), which is the ratio of FI to weight gain, or its inverse, known as feed conversion efficiency [32,33]. Residual/regression traits include RFI/net FI, which is the difference between actual and predicted DMI [34].

### 2.1. Feed Conversion Ratio/Feed Conversion Efficiency (FCR)

The most commonly used FE index is FCR, or its inverse, referred to as gross FE. In meat production systems, outputs are commonly assessed by the weight gain of growing animals. The FCR is determined by dividing FI by weight gain over a specific period of growth. Gross FE is calculated as the ratio of weight gain to FI. Alternatively, the output can be defined as lean tissue, and the percentage of lean tissue gain to FI can be used to measure efficiency. The period of growth over which FCR is measured is typically defined on a time-constant basis, where growth and FI are measured between two specific time points. Other alternatives that have been used to account for maturity patterns or scale in the measurement include a weight-constant basis (calculating feed required for growth from one weight to another). Another approach is a maturity-constant basis (measuring feed and weight gain from one stage of maturity to another or from a specific subcutaneous fat depth to another; e.g., [35,36,37]). However, the practical application of weight-constant or maturity-constant measurements to many animals is challenging due to the increased data requirements. Numerous studies have provided evidence for the phenotypic and genetic correlations between FCR and production traits in beef cattle. For instance, Archer et al. [38] summarized four studies that revealed significant negative genetic correlations (−0.61 to −0.95) between growth rate and FCR. This indicates a strong relationship between the rate of growth and the efficiency of converting feed into weight gain in beef cattle.

Heritability estimates for FCR in cows have shown considerable variation in previous studies, ranging from 0.00 [39] to 0.38 [40]. In growing animals, the minimum heritability for FCR was 0.06, with 0.46 being the maximum. The heritability estimates for FCR in mature cows ranged from 0.05 to 0.32 [38]. Torres-Vázquez et al. [41] estimated the FCR of Australian Angus beef cattle to be 0.20 ± 0.06. Novo et al. [42] recorded heritability of 0.09 ± 0.05 in Senepol heifers. Compared with other traits estimated by regressions using residual gain (RG), residual intake gain (RIG), and RFI, FCR was found to be less heritable. Moreover, FCR had genetic correlations of −0.21 ± 0.27, −0.83 ± 0.13, −0.30 ± 0.32, 0.11 ± 0.27, 0.09 ± 0.25, 0.46 ± 0.29, and −0.38 ± 0.28 with BW, ADG, DMI, rib-eye area (REA), backfat (BF), intramuscular fat (IMF), and carcass conformation score (CCS), respectively. Phenotypic correlations recorded in the same study were −0.05, −0.80, 0.14, −0.01, −0.07, −0.10, and 0.00 with BW, ADG, DMI, REA, BF, IMF, and CCS, respectively [42]. Smith et al. [43] recorded high phenotypic correlations between FCR and three measures of RFI (DMI adjusted for production, RFI_P_; DMI adjusted for ultrasonic backfat thickness, RFI_BF_; DMI estimated using the NRC net energy equations, RFI_NRC_) to be 0.68, 0.68, and 0.71, respectively. Santana et al. [44] reported that FCR had genetic correlations of 0.10, 0.95, and −0.87 with DMI, RFI, and RIG, respectively. The phenotypic correlations reported were 0.17, 0.34, and −0.46 with DMI, RFI, and RIG, respectively.

The strong genetic correlations between gross FE and production traits suggest that selecting moderate to high heritability traits such as growth rate can improve gross FE without directly measuring FI [33]. Research by Mrode et al. [45] on Hereford cattle showed a more significant improvement in lean FCR through selection for lean growth rate than direct selection for FCR alone. However, improving the FCR may not enhance overall system efficiency or profitability. Higher growth rates in genotypes often lead to increased mature cow weights and higher feed requirements for the cow herd, which can offset gains in growth efficiency. Maturity patterns influence the FCR of growing animals, and if increased feed requirements of the breeding herd negate the improvements in growth efficiency, production system FE may remain unchanged. Nevertheless, there may be economic efficiency gains if the feed value for the slaughter generation surpasses that for the breeding herd.

One limitation of FCR is its strong correlation with FI and growth rate. Consequently, focusing on the feed:gain ratio during selection may result in increased maintenance needs for animals with larger mature weights. Animals with similar FCRs can exhibit significant variations in their growth rates and FI. Selecting based on ratio traits makes predicting changes in individual traits in future generations challenging to achieve with high accuracy [43]. Another limitation is the typical avoidance of ratio traits in breeding programs. Conventional breeding models often assume additive genetic effects, which may not accurately capture the complexities of ratio traits that involve non-additive genetic effects (as they do not follow the assumptions in most models) [46]. More so, interpreting and understanding ratio traits can be challenging for farmers. Ratio traits often have complex relationships with other traits and can interact differently under various environmental conditions. This complexity makes it difficult for farmers to assess the practical implications and trade-offs of incorporating ratio traits into their breeding goals [47]. Numerous studies have examined the effects of increased mature size on FE in beef production systems [48,49,50,51]. The consensus from these studies, particularly in maternal breeds, is that an increase in mature size has minimal impact on production system FE. The FCR remains a useful measure of efficiency for scenarios involving only growing cattle or specific breeding purposes such as terminal sires. However, it is unlikely to correlate with the FE of beef production systems when accounting for the requirements of the breeding herd [38].

### 2.2. Residual Feed Intake (RFI)

Koch et al. [34] introduced the concept of RFI to account for the impact of both weight maintenance and weight gain on the feed requirements of growing cattle. They proposed adjusting FI based on BW and weight gain, separating it into two components: (1) the expected FI for a specific level of production and (2) a residual portion that allows for the identification of animals that deviate from their expected level of FI. Overall, efficient animals typically have lower (negative) RFIs. RFI is an index not influenced by the production traits used to calculate expected FI. Unlike in FCR, this allows for comparisons between individuals with different production levels during the measurement period. Because RFI is independent of production, researchers such as Korver [33] proposed RFI as a good indicator of efficiency, reflecting variations in metabolic processes. RFI may also be correlated with the overall FE of the production system, as it adjusts for production during the measurement period. This adjustment reduces the complexities associated with interpreting gross FE.

Although RFI is phenotypically unrelated to the production growth rate and BW in growing cattle [10,52,53,54,55], it shows certain relationships under specific conditions. When RFI is calculated by phenotypic regression of production on FI, the resulting efficiency measure is not necessarily genetically independent of production. Some studies have found genetic independence between RFI and production traits [52,56]. However, genetic variation in RFI may include genetic variation in production traits. It may also reflect inherent relationships between FI and production. In this context, Kennedy et al. (1993) suggested a method to obtain a measure of efficiency that is genetically independent of production. They proposed calculating genotypic RFI using genetic (co)variances rather than the phenotypic (co)variances used in the phenotypic regression approach. Genotypic RFI is genetically independent of production and thus may be more likely to reflect genetic differences in inherent relationships between FI and production. Production differs between studies; for instance, Nkrumah et al. [57] found RFI to be genetically independent of ADG and BW but showed a phenotypic correlation with ADG (r = −0.21; *p* < 0.05). In comparison, Ceacero et al. [58] found unfavorable genetic correlations between RFI, RFI adjusted for backfat thickness (RFI_b_), and RFI adjusted for backfat and rump fat thickness (RFI_sf_) with weight at selection (WS) (0.17, 0.23, and 0.22), BF (0.37, 0.33, and 0.33), and RF (0.30, 0.31, and 0.32).

However, knowledge of genetic relationships between FI and production is required to calculate genotypic RFI or predict correlated responses in FI and production to selection based on phenotypic RFI. Most animal production systems have limited information on these genetic relationships. In summary, it seems that selecting for RFI is better than selecting for FCR. The reason is that this approach decreases FI in both young and adult cattle without affecting growth performance or increasing cow size [55].

Multiple studies have demonstrated that RFI and FCR exhibit moderate heritability across beef cattle breeds [53,56,57,59]. However, Sainz et al. [60] reported low to moderate heritability (0.19 ± 0.017) for RFI in purebred Nellore cattle and suggested that long-term selection to reduce RFI can be beneficial for yearling weight and carcass quality traits. Similar heritability (0.21 ± 0.02) was also reported by Brunes et al. [61] in Nellore Cattle. RFI adjusted for fat, with higher heritability of 0.44, has been reported to promote genetic gains, resulting in efficient food use without negative changes in carcass traits [62]. Genetic selection can reduce maintenance energy needs and RFI, lowering feeding costs while preserving product quality and output.

Archer and Arthur [54] reported a strong correlation (>0.90) between post-weaning RFI in heifers and RFI measured in the same females as mature cows. This finding implies that selecting for enhanced post-weaning RFI can yield offspring that exhibit efficiency across all industry sectors. These studies have also shown a correlation between RFI and FCR, ranging from 0.45 to 0.85. Therefore, selecting for improved RFI will likely lead to genetic changes in FCR.

### 2.3. Residual Average Daily Gain (RADG)

A recent approach to measuring FE is residual average daily gain (RADG). RADG is calculated by subtracting the predicted ADG from the actual ADG. It is worth noting that a positive or high RADG value is desired as it indicates a more significant gain. The American Angus Association (AAA) has developed this method and created an expected progeny difference (EPD) to assess RADG. The RADG EPD is a product of research projects funded by the Angus Foundation and the American Angus Association [63]. These projects, performed by the University of Illinois, North Carolina State University, and Iowa State University, have collected individual intake data over several years. The RADG EPD provides a comprehensive approach to identifying cattle that excel at converting a specific quantity of feed. By combining the individual intake data with genomic information on DMI, Angus producers can access a nearly real-time selection of more feed-efficient genetics. According to the AAA [64], a comprehensive genetic evaluation can quickly determine RADG without requiring a feed test. This evaluation incorporates anchor traits, such as weaning weight, post-weaning gain, subcutaneous fat thickness, calf DMI, and DMI genomic values. These genetic values, combined with animal ADG and fat measurements, serve as predictors for an animal’s RADG potential.

RADG has a moderate heritability (0.31 to 0.41), making it practical for improving FE in cattle production systems. Freetly et al. [65] reported heritability of 0.21 ± 0.11 in heifers and 0.14 ± 0.10 in cows in a study that compared the relationship between FI in growing heifers and mature cows. In this study, heifer RADG had genetic correlations of −0.86 ± 0.40, 0.05 ± 0.30, −0.13 ± 0.28, and 0.52 ± 0.11 with heifer RFI, cow ADG, cow average daily dry matter intake (ADDMI), and heifer ADG, respectively. Cow RADG had a heritability of 0.14 ± 0.10 and genetic correlations of −0.86 ± 0.57, 0.31 ± 0.46, −0.16 ± 0.43, 0.50 ± 0.16, 0.33 ± 0.30, and 0.20 ± 0.26 with cow RFI, heifer RADG, heifer RFI, cow ADG, heifer ADG, and heifer ADDMI, respectively. The genetic correlations between RFI and RADG within heifers and cows were both −0.86, as these two efficiency measures have opposite directional preferences. The genetic correlations between heifers and cows for RFI and RADG were 0.41 ± 0.36 and 0.31 ± 0.46, respectively [65].

However, it is essential to consider that RADG and FCR are suitable for feedlot animals but pose challenges for cow-calf producers. According to the AAA [64], selecting based on RADG or FCR may result in larger, heavier cows with higher nutrient requirements, which is not advantageous for cow efficiency or the production system. The ability to provide EPDs for RADG gives Angus producers the power to make accurate decisions [64]. Making selection decisions based on EPDs for RADG may improve FCR and reduce RFI without compromising growth or carcass quality, suggesting that enhancing FE should not compromise animal productivity or meat quality.

Table 1 summarizes heritability estimates of FE traits across several breeds and populations for growing animals, and heritability estimates for mature cows are summarized in Table 2.

### 2.4. Maintenance Efficiency

Another method of measuring FE traits involves dividing the feed consumed into portions required for maintenance and production (growth, lactation, and gestation). One example is maintenance efficiency, which calculates the ratio of FI used for maintenance (actual intake minus predicted intake for growth) per unit of metabolic body size, BW^0.75^ [96]. Maintenance requirement is the feed energy needed for an animal to maintain a constant BW [38]. On the other hand, maintenance efficiency is the ratio of BW to FI when there is no change in BW. In a typical beef breeding herd, the energy required for maintenance constitutes 60–75% of the total energy needs of individual breeding cows [15,38]. Additionally, it is estimated that the cow herd utilizes 65–85% of the energy required for beef production [15,38], depending on the production system. However, these estimates are based on temperate beef production systems, and extensive pastoral systems in northern Australia might differ. These systems may exhibit different proportions of feed utilization due to lower reproductive rates and higher ages at slaughter.

Nevertheless, the cost of maintaining breeding cows is crucial in determining the efficiency and profitability of beef production systems. However, measuring maintenance efficiency presents practical challenges. Measuring maintenance efficiency in growing animals is unrealistic because weight stasis is not achieved as young animals are still experiencing growth. Proxy measures such as fasting heat production have been proposed and used. However, studies have shown that an animal’s growth trajectory can significantly influence maintenance efficiency. This suggests that measurements should account for factors beyond just maintenance requirements [38]. To accurately measure maintenance requirements, animals need to be kept at a constant live weight, which may take as long as two years in beef cattle [97]. Such measurements require significant resources and costs.

### 2.5. Partial Efficiency of Growth

Partial Efficiency of Growth (PEG) measures weight gain compared to the feed consumed after accounting for the animal’s maintenance needs (AFI minus predicted feed for maintenance). These maintenance needs can be determined using feeding tables or metabolic studies. However, both methods have their limitations. Feeding tables, which are standardized references of dietary requirements, assume that the efficiency of feed use for maintenance is consistent. However, this assumption may not be accurate, especially in forage-based diets where nutrient profiles vary more significantly than in concentrate-based feeds. Additionally, nutritionists often use a dynamic mechanical model (alongside empirical models) that estimates nutritional requirements based on an animal’s changing live weight and condition. Metabolic models, which dynamically estimate nutritional requirements, offer an alternative to feeding tables but have limitations of their own. These models are complex and require extensive data, making them difficult to apply in practical settings with large or diverse groups of animals. Additionally, they rely on static parameters that may not fully adapt to rapid changes in an animal’s condition, such as stress or illness. Both feeding tables and metabolic models can benefit from incorporating nutrient analysis of the actual diet to improve accuracy, especially in forage-based diets where nutrient content can fluctuate widely. Thus, the PEG fails to capture the inherent variations in maintenance-related energetic efficiencies. However, PEG seems to have an advantage over FCR as a measure of FE. Grion et al. [67] recorded heritability of 0.14 ± 0.07 in Nellore cattle when collective pens were used and an estimate of 0.25 ± 0.09 without collective pens. They also found genetic correlations of −0.23 ± 0.18, −0.67 ± 0.11, −0.29 ± 0.21, and −0.37 ± 0.16 with weaning weight adjusted to 210 days of age (W210), DMI, ADG, and metabolic BW (BW^0.75^), respectively. In addition, phenotypic correlations of −0.13 ± 0.03, −0.54 ± 0.03, 0.17 ± 0.04, and −0.24 ± 0.04 between PEG and W210, DMI, ADG, and BW^0.75^, respectively.

Nkrumah et al. [57] also observed a marked genetic correlation between PEG and DMI (−0.51) in Canadian crossbred heifers. Fan et al. [68] estimated a genetic correlation between ADG and PEG of −0.57 and −0.62, whereas Nkrumah et al. [57] obtained a genetic correlation of 0.55. Nkrumah et al. [98] reported a phenotypic correlation of −0.89 and −0.83 with RFI and FCR, respectively. They also recorded phenotypic correlations between PEG and carcass traits: −0.30, −0.25, −0.27, 0.24, and −0.25 with backfat gain, ultrasound backfat, grade fat, lean meat yield, and yield grade, respectively. Carstens and Tedeschi [96] recorded Pearson’s correlation of −0.77, 0.27, −0.52, −0.15, −0.10, 0.20, −0.25, −0.57, and −0.87 between PEG and FCR, a dry matter required (DMR), DMR to ADG ratio (R: G), BF, REA, ADG, initial body weight (iBW), DMI, and RFI in growing calves. In finishing calves, the correlation recorded between PEG and FCR, DMR, R: G, BF, REA, ADG, iBW, DMI, and RFI was −0.79, 0.27, −0.52, −0.38, 0.02, 0.11, −0.38, −0.64, and −0.84, respectively. In both growing and finishing calves, PEG showed strong correlations with FI, with values of −0.57 and −0.64, respectively [96]. As mentioned above, the phenotypic correlations between ADG and PEG (0.24) are significantly lower than between ADG and FCR (−0.63). Grion et al. [67] recommend that PEG and RFI provide the best responses to selection for reduced DMI, proportional to high genetic gains in growth traits. This is applicable in the context of 2-stage selection schemes, which include preselection during the post-weaning period.

### 2.6. Cow/Calf Efficiency

Cow/calf efficiency is another alternative to assess the efficiency of the beef production system [69]. This index is designed to assess a cow’s ability to produce marketable calves with minimal input costs while ensuring herd health and profitability. There are multiple methods for calculating this index, depending on the available information. The simplest approach is to calculate the ratio of the weight of the weaned calf to the cow’s weight. This method is already employed by some beef cattle genetic evaluation programs worldwide. A second method is recommended for assessing the metric at the herd level. This involves calculating the total weight of the weaned calves divided by the number of cows that entered the breeding season. The third method focuses on FE and requires consideration of the total feed intake (TFI) of the cow and her offspring throughout an entire production cycle. This cycle typically spans from the weaning of one calf to the weaning of the next. By comparing the TFI over the production cycle with the weight of the weaned calf, cow/calf efficiency is expressed as the ratio of kilograms of calf weaned per kilogram of feed consumed. This method captures the efficiency of the cow/calf unit in terms of both biological and economic aspects, as it considers the FI and production of the breeding herd and the pre-weaning phase of the progeny. It reflects the efficiency of animals in a realistic production state. However, cow/calf efficiency does not account for the FI of the slaughter generation from weaning to slaughter or the replacement animals entering the cow herd. Additionally, it does not factor in the output from the sale of culled cows.

Nonetheless, since the feed utilized for post-weaning growth represents a relatively small portion of total feed used in beef production in many systems, cow/calf efficiency may reasonably indicate production system efficiency [38]. While cow/calf efficiency may be more strongly associated with production system FE than other efficiency indices, its practical application and use in genetic studies are challenging. Measuring cow/calf efficiency entails significant costs and effort and is highly dependent on the nutrient profile of the ration, especially forage quality and the amount of supplemental feeds needed to meet maintenance requirements. However, cow/calf efficiency can be valuable as an indicator of the extent of phenotypic variation in the FE of production systems.

Research utilizing the cow/calf unit as a measure of efficiency has provided evidence of substantial variation in cattle production’s efficiency. In two separate years, the coefficient of variation for cow/calf efficacy among 33 Hereford × Angus heifers studied by Shuey et al. [69] was 6% and 7%. The calf gained between 6.0 and 8.2 g at weaning for every megajoule of metabolizable energy the cow and calf consumed [38,69]. Similarly, an Australian study discovered that even within the same herd and genetic background, cow/calf efficiency varied widely, with some cows requiring up to 50% less feed per kilogram of weaned offspring than others [38]. Jenkins and Ferrell [70] compared British and European strains at differing nutrition levels to demonstrate that genetics influence the feed utilization efficacy of a production system. The study recorded individual cow consumption and daily feed allowance adjusted for refusal weekly. They discovered a significant relationship between genotype and feeding level, with higher-yielding genotypes, defined by weight at weaning, achieving their optimum efficiency at elevated feeding levels, while lower-yielding genotypes at reduced feeding levels. In addition, as the quantity of food consumed increased, the efficiency of the latter group declined. This genotype-feeding level interaction was primarily driven by differences in reproductive rate, highlighting the need to optimize production by matching genotype to the environment (in this case, feed availability). However, Jenkins and Ferrell [70] did not specify how much variation in FE is not attributable to differences in reproductive rate between or within breeds.

## 3. Data Collection Technologies

Traditional ways of collecting information on FI in dairy and beef cattle are usually labor-intensive. These methods often involve time-consuming processes such as watching them directly, recording videos over time, and manually measuring feed delivery and feed refusal. However, these methods are limited, mainly when used for a long time or with many animals. Also, much of the research has been completed in environments that may not accurately represent how animals behave in pasture conditions, in groups such as pens, tied stalls, or feed bunks with limited access. Because there’s a growing need for a large database that includes FI as a trait for breeding programs and for studying precision livestock farming, new tools are being developed to measure FI better.

One method includes using electronic scales and radio frequency identification (RFID) antenna positioned inside feeding stalls. This electronic scale is the oldest and one of the most direct methods used in group housing and feedlots [71]. The scale is placed inside a feeding station to measure the precise amount of feed consumed by each animal during every meal at each designated feed bin. Each farm determines the appropriate quantity of electronic scales to use along a feeding lane by the number of animals. Several companies have developed systems for this purpose, including the Calan Broadbent Feeding System, the Controlling and Recording Feed Intake System, the GrowSafe System (recently acquired by Vytelle, a precision livestock company), Intergado Efficiency, and the Roughage Intake Control System. Many researchers have also evaluated the efficiency of these systems [71,72,73,74,75,76,77,78]. Nevertheless, these systems are rarely extensively used in commercial operations due to their exorbitant cost and the substantial cleaning and maintenance requirements. Additionally, some systems exercise control over the data they gather, and the user is not provided with a clear understanding of how it is managed.

**Figure 1:**
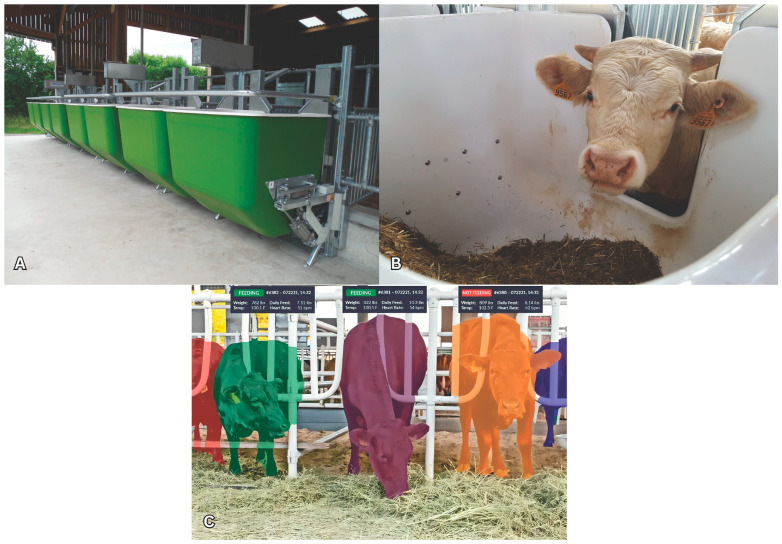
Example of equipment used to monitor and record feed intake in cattle. (**A**) The feeding lane is equipped with multiple electronic scales for individual feed intake monitoring—adapted from Biocontrol (https://biocontrol.no/products-2/controlling-and-recording-feed-intake/, accessed on 6 November 2024). (**B**) Close-up of a feed intake system with individual animal identification—copyrights sourced from Biocontrol (https://biocontrol.no/products-2/controlling-and-recording-feed-intake/, accessed on 6 November 2024). (**C**) Visual monitoring of feeding behavior using color-coded overlays on each animal to track feeding status and health parameters in real-time—copyrights sourced from Miguel Ángel Cabrera Miñagorri/Pipeless (https://www.pipeless.ai/industries/cattle-raising, accessed on 6 November 2024).

Other studies have used affordable cameras and computer vision algorithms to develop innovative methods for accurately measuring FI. The camera is often positioned above the feed bunk. Various techniques are utilized to visually represent the three-dimensional (3D) location of the surface captured by the camera. One of these methods uses structured light illumination (SLI) and time of flight [79] using a camera and light projector. Light patterns are projected onto the observed area. SLI scanned the 3D structure of feed to determine its volume and weight in a bin before and after feeding [80]. The SLI method significantly differs between estimated image weight and real values on 272 piles in a lab [80]. Only 72% of findings were within 814 g of the comparison between image-estimated mass and scale-measured mass. Unfortunately, SLI systems only operate indoors, shielded from sunlight, because they require regulated lighting, tuning, and shade. The system also needs eight cameras per heap, making it impractical. Another challenge is that each time the moisture content of the diet or ration ingredients is changed, the camera algorithms must be recalibrated. Figure 1 shows some examples of equipment used to record FI.

Another method uses calibrated stereo cameras to monitor FI via triangulation and point disparity analysis to derive depth information. Bloch et al. [82] used photogrammetry to quantify feed mass and volume, producing a 3D model of several ration heaps from various angles. The procedure was tested with 125 and 60 ration piles in the lab and cowshed. The estimated inaccuracy for feeding piles up to 7 kg was 0.483 kg in the lab. The cowshed experiment had a standard deviation of 0.44 kg and a total error of 1.32 kg for ration piles up to 40 kg. The colored markers used for point cloud processing may not be viable in a cowshed on a working farm since dirt might distort their colors and dislodge them from the floor and walls owing to tractor use and ventilation.

FI may also be monitored with red, green, and bluedepth (RGB-D) cameras and infrared sensors. These cameras provide depth data for each RGB pixel via an infrared (IR) or near-IR projector-based depth sensor. This 3D data-gathering method has been utilized in research and industry to evaluate object surfaces [83]. Indoor, outdoor, and open cowshed RGB-D feed intake techniques and algorithms have been developed [79,84,85]. An RGB-D camera and deep learning system mitigated sunlight’s influence on an open cowshed’s IR scanner [85]. The device correctly assessed one meal’s FI with a mean absolute error of 0.127 kg per meal from 0 to 8 kg. When integrated with eating behavior sensing, developed FI techniques and algorithms may be enhanced [86].

Precision phenotyping in livestock has been revolutionized with recently developed technologies for welfare, prediction, and diagnosis [87,88]. One of which is 3D cameras for estimating FI and BW in commercial herds. Unlike scale-based methods, the Cattle Feed Intake System (CFIT; [79,89,90,99]) uses 3D camera records and artificial intelligence to forecast individual DMI and BW. The CFIT’s barn-mounted 3D cameras can recognize cows and use artificial intelligence algorithms to collect herd DMI and BW [91,92,93]. This technique has been used in dairy cattle to forecast FI and BW for each cow during the lactation and across all lactations. Importantly, 3D cameras on the barn roof do not interfere with animal eating as feed bins do. It addresses limited FI and efficiency records in early lactations and can be used in beef cattle production systems. More recently, Lassen et al. [89,94] and Manzanilla-Pech et al. [89,94] obtained FI data from 3D cameras on 17 Danish commercial dairy farms from 2019 to 2021. The cows were videotaped while eating using cameras above the roof-mounted feed bunk [79,99]. A radio frequency identification reader scanned the ear tags, and the 3D camera used time-of-flight technology. AI-based algorithms identify cows and transform 3D pictures into phenotypes (DMI and BW). Each cow is assigned records for FI after visiting the feed bunk. From each specific visit, five variables are stored: the ID of the cow, the placement in the barn, the meal start time, the meal end time, and the total amount of feed consumed. Lassen et al. [89] reported repeatability estimates ranging from 0.62 to 0.65 for daily FI and from 0.83 to 0.88 for BW measured as a weekly average.

Technological methods for monitoring FI in cattle offer significant advancements over traditional approaches but are expensive, and practical limitations still hinder large-scale adoption. For instance, traditional methods such as direct observation and manual measurements are labor-intensive, time-consuming, and lack scalability, making them unsuitable for large-scale operations or prolonged studies. Electronic scales with RFID integration provide high accuracy and automation but are expensive for many commercial operations. Additionally, these systems require substantial maintenance and often involve proprietary data handling, which can restrict user control. Emerging technologies, such as 3D cameras and RGB-D sensors, provide innovative solutions through non-invasive, continuous monitoring with artificial intelligence-enhanced precision. The 3D camera systems, such as CFIT effectively estimate FI and BW without interfering with animal behavior, though they involve relatively high initial setup costs. On the other hand, techniques such as photogrammetry and SLI are cost-effective alternatives for smaller-scale implementations but are less practical due to their sensitivity to environmental factors such as lighting conditions and dirt. While the upfront investment for advanced systems is substantial, their long-term cost-effectiveness is more favorable due to reduced labor needs and improved data accuracy compared to traditional methods. These considerations underscore the trade-offs between initial investment, operational efficiency, and scalability in selecting an FI monitoring approach.

## 4. Genetic Selection

Understanding the genetic relationships between FE and various physiological and production traits is crucial for optimizing livestock management and breeding strategies. FE is a complex trait influenced by multiple factors, including maintenance requirements, growth, carcass traits, and body composition. In this section, we will explore the intricate connections between FE and these key aspects, as well as the physiological and extra-physiological considerations that contribute to its variation. By examining these relationships, we aim to provide a comprehensive framework for improving feed efficiency in beef cattle production systems.

### 4.1. Genetic Relationships Between Feed Efficiency and Growth and Carcass Traits

Although FI and other growth traits have been widely used in FE studies, some studies provide insights into their relationship with carcass traits. In a study evaluating FE traits in Japanese Black cattle over three separate periods, Takeda et al. [100] found that RFI exhibited moderate genetic correlations with FI (0.53 to 0.63) and low genetic correlations with daily gain (0.00 to 0.08). On the other hand, the residual BW gains showed moderate genetic correlations with daily gain (0.33 to 0.61) and low correlations with FI (−0.14 to 0.27). Among these FE traits, the strongest correlations were observed with FCR, with absolute values ranging from 0.63 to 0.96. Specifically, residual intake gains (RIGs) exhibited the highest correlations (ranging from −0.84 to −0.96) compared to the other FE traits. In the same study, carcass traits, including carcass weight (CW), ribeye area (REA), subcutaneous fat thickness (SFT), rib fat thickness (RT), and marbling fat score (BMS), were evaluated at 21 months of age. The heritability estimate for BMS was the highest at 0.77, while moderate heritability estimates were observed for CW, REA, RT, and SFT at 0.66, 0.59, 0.51, and 0.57, respectively. During different fattening periods, the three FE traits exhibited weak correlations with all carcass traits, ranging from −0.05 to 0.19 for RFI, 0.02 to 0.31 for RG, and −0.11 to 0.20 for RIG. In the first half of the fattening period, RG and RIG showed significant positive genetic correlations with CW and REA, but these correlations were not significant in the latter half.

Arthur et al. [52] conducted a study on Angus cattle and reported heritability estimates of 0.39 for FI, 0.28 for daily gain, and 0.29 for FCR. Similarly, Hoque et al. [101] and Retallick [102] studied Japanese Black cattle and found heritability estimates of 0.36 for FI and 0.38 for FCR. Retallick [102] also discovered high genetic correlations between FCR and residual gains (RG) at −0.97 and residual intake gains (RIG) at −0.95. Based on these findings, selecting cattle with high RIG may result in reduced FI and increased BW gain. Elolimy et al. [103], another study that evaluated the association between RFI and carcass traits in Red Angus cattle, arrived at some interesting findings. Significant differences were found in carcass traits based on the grouping of RFI, with the most efficient animals showing higher hot carcass weight, kidney, pelvic, heart fat, and ribeye area than the least efficient animals (*p* ≤ 0.05). However, no significant differences were observed in carcass traits when considering the interaction between RFI and sex (*p* > 0.10).

Santana et al. [44] found low genetic correlations between RIG and ribeye area and subcutaneous fat thickness in Nellore cattle (0.02 and −0.03, respectively). Retallick [102] reported low genetic correlations between RIG and carcass weight, loin eye area, backfat, and marbling score (ranging from −0.09 to 0.20). Ceacero et al. [58] also observed favorable genetic correlations between RIG and carcass traits, with values of 0.16 for loin eye and −0.38 for subcutaneous fat thickness. These studies suggest that selecting individuals with high RIG can improve overall cattle performance without adversely affecting carcass traits. Regarding the genetic relationships between RFI and carcass traits in Japanese Black cattle, previous studies have found similar patterns except for the relationship with BMS. Hoque et al. [101] reported a correlation of −0.59 between RFI and BMS, while Inoue et al. [104] reported a correlation of 0.51. This discrepancy may be due to differences in population size or the definition of RFI. Hoque et al. [101] studied 514 bulls from 22,029 progenies, while Inoue et al. [104] studied 863 bulls from a population of 4578 animals. In conclusion, selecting animals with high RIG is recommended to improve FE and BW gain without negatively impacting carcass traits. On the other hand, selecting animals with low RFI can lead to improvements in FE alone [100].

A more recent study conducted a genetic evaluation of FI in beef cattle as a relationship between FI in growing heifers and mature cows [65]. In the study, 687 heifers and 622 5-year-old cows were used, and the heritability of ADDMI was estimated to be 0.84 ± 0.12 in heifers and 0.53 ± 0.12 in cows. The heritability of ADG was estimated to be 0.53 ± 0.12 in heifers and 0.34 ± 0.11 in cows. The genetic correlation between heifer and cow ADDMI was 0.84 ± 0.09, indicating a strong genetic relationship. Similarly, the genetic correlation between heifer and cow ADG was 0.73 ± 0.19. The heritability of RFI was estimated to be 0.25 ± 0.11 in heifers and 0.16 ± 0.10 in cows. RG’s heritability was 0.21 ± 0.11 in heifers and 0.14 ± 0.10 in cows. The genetic correlations between RFI and RADG were −0.86 in heifers and −0.86 in cows. This indicates that the two measures of efficiency operate in opposite favorable directions. The genetic correlations between heifers and cows for RFI and RADG were 0.41 ± 0.36 and 0.31 ± 0.46, respectively. These findings suggest that both FI and ADG are heritable traits and exhibit genetic correlations between heifers and cows. Consequently, selecting for decreased FI and ADG in growing animals is likely to have similar effects on mature cows. However, further studies are needed in this context, especially focused on different breeds [65].

### 4.2. Genetic Relationships Between Feed Efficiency and Maintenance Requirements

Limited information is available regarding genetic variation in maintenance efficiency within breeds due to the challenges and costs associated with measuring it in a sufficient number of cattle. However, two studies using twin pairs have found genetic variation in maintenance efficiency within specific breeds. Taylor et al. [97] observed a genetic coefficient of variation of 6.4% in Ayrshire twins, suggesting genetic variability within the Ayrshire breed. Archer et al. [38] measured heat production in monozygotic twin pairs from different breed combinations and found significant variation in estimated maintenance requirements, with heritability estimates ranging from 0.17 to 0.71 at various ages. It is essential to exercise caution when extrapolating these findings to adult cattle, but variation in maintenance efficiency likely exists within breeds.

#### 4.2.1. Physiological Basis for Variation in Feed Efficiency

Three compelling reasons exist to investigate the mechanisms underlying differences in reported FE. Understanding the physiological basis of disparities in FE enables us to anticipate potentially correlated responses to selection. While this information cannot be used to predict the genetic effects of selection, it can assist researchers in identifying responses associated with selection. Understanding the physiological underpinnings of FE variation could lead to identifying traits that are less expensive to detect than FI and efficiency and could be used as proxies during the selection. In conclusion, understanding the physiological causes of variation in FE could lead to the development of novel, non-genetic methods for altering the metabolic rate of cattle and thereby improving their FE.

#### 4.2.2. Distribution of Nutrient Demands

**Figure 2:**
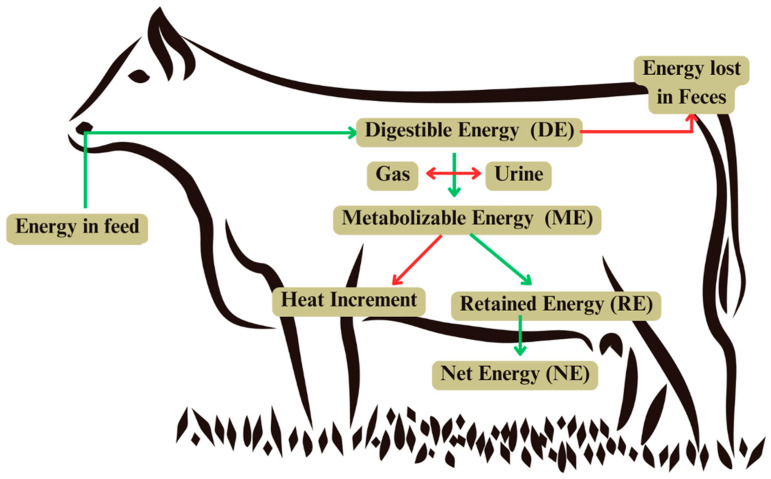
Summary of the energy flow in cattle.

Typically, the animal fodder is categorized into two main types: maintenance and production requirements. Maintenance requirements focus on the energy needed to sustain basic physiological functions such as breathing, blood circulation, and thermoregulation, while production requirements encompass the additional energy needed for growth, reproduction, or lactation [33]. The energy in feed is quantified as gross energy (GE), which represents the total chemical energy within the feed. However, not all this energy is utilized by the animal; a portion is lost in feces, representing the undigested portion. The energy remaining after fecal losses is termed digestible energy (DE). From DE, further losses occur as gases and urine, leading to metabolizable energy (ME), which is the energy available for the animal’s metabolic processes. The conversion of ME into useful forms of energy involves further energy dissipation as heat, known as the heat increment, which includes energy lost during digestion and nutrient metabolism. The energy remaining after accounting for the HI is referred to as net energy (NE). The NE is divided into energy used for maintenance (e.g., sustaining body functions) and retained energy, which supports production activities such as growth or milk synthesis [105]. A summary of the energy flow in cattle is shown in Figure 2.

Adult cattle fed forage-based diets generally require over 50% of their total dietary consumption for body maintenance, whereas growing cattle typically require more than 40%. Several physiological and biochemical mechanisms contribute to the high demand for maintaining homeostasis and may affect FE. According to Bottje and Carstens [106], mitochondria are responsible for producing around 90 percent of the oxygen that is found in a cell. Kolath et al. [107] studied the respiratory control ratio (RCR) in the *longissimus* muscle tissue of steers, which indicates the degree of coupling between oxidative phosphorylation and respiration, a measure of how efficiently electrons are transferred. They observed that steers classified as low RFI had RCR significantly greater than that of steers classified as high RFI. In the study performed by Fitzsimons et al. [108], tissue samples from young beef bulls were analyzed. Citrate synthase activity, used as an indicator of mitochondrial number, showed no correlation between RFI status and the number of mitochondria in either the muscle or liver tissue.

A study of bovine liver tissue performed by Lancaster et al. [109] indicates, on the other hand, that energetically inefficient steers have a lower level of ADP control of oxidative phosphorylation than feed-efficient steers. The acceptor control ratio (ACR; ratio of state 3: state 2 respiration), which indicates the respiratory rate within the mitochondrion, was higher in low-RFI cattle [109]. This finding was also observed in steer progeny of sires with divergent RFI [110]. According to Ramos and Kerley [111], lymphocytes obtained from low-RFI steers contain larger amounts of mitochondrial complex I than those obtained from high-RFI steers, which suggests that the former generate more ATP. Studies assessing differential mRNA expression of genes linked with oxidative phosphorylation in beef cattle’s muscle or liver tissue divergent for RFI have found inconsistent results at the cellular transcript level [112,113].

Furthermore, it has been postulated that variations in stress reactions between animals classified as high and low RFI are one of the processes that contribute to the observed disparities in energy efficiency. These hypotheses are based on evidence that reveals differences in stress responses between animals classified as high and low RFI. Recent research by Kenny et al. [114] showed that low-RFI Simmental heifers tended to have lower sensitivity to exogenous adrenocorticotropic hormone. This finding suggests that RFI status may be connected to the hypothalamic-pituitary-adrenal axis function in cattle [115]. Cortisol concentrations in high-RFI and low-RFI Limousin heifers were found to be identical in a recent investigation on the hormonal responses to a corticotropin-releasing hormone challenge [116]. In the study that was carried out by Munro et al. [117], plasma cortisol levels were not measured; however, it was noted that low-RFI heifers had a significantly elevated heart rate in response to an acute stressor. This was the finding that stood out the most.

#### 4.2.3. Body Composition

Researchers have shown that fatter cattle have fewer maintenance needs than leaner livestock of the same live weight, and this effect holds across a wide range of animal species [105]. It is estimated that the ratio of retained energy (RE) to expended energy is 0.88 for protein synthesis and 0.81 for lipid synthesis, indicating that protein production is more energy efficient. Once proteins have been synthesized, they undergo constant degradation and reconstruction. Due to this “turnover”, protein is maintained at a lower efficiency than fat (0.4 for protein versus 0.70–0.75 for fat; [118]). Owens et al. [119] found that fat accretion had an average efficiency of 76% (heat loss of 24%), whereas protein accretion was only 47% (heat loss of 53%). Therefore, dietary requirements are determined by considering both body and gain composition.

Recent studies indicate that lines of Angus cattle selected for and against the yearling growth rate exhibit comparable differences in protein turnover rates (Oddy et al., 1998, as cited in [38]). Due to differences in protein turnover rates, there may be an inherited component to the variation in the quantity of feed energy required for maintenance and growth. Larger percentages of subcutaneous fat, as seen in beef breeds, were found by Thompson et al. [105] to be associated with lower maintenance costs than larger percentages of visceral fat, as shown in dairy cow breeds. This finding may partially explain differences in maintenance efficiency between dairy and beef breeds. Dairy breeds have higher energy and weight balance maintenance needs than beef breeds, according to research by Solis et al. [120]. They hypothesized that this may be due to differences in fat distribution throughout the body. In addition, beef breeds’ maintenance energy needs reflect their lean body mass and subcutaneous fat. In contrast, dairy breeds’ maintenance energy needs reflect their lean body mass, internal fat, and vital organ mass. These physiological differences accounted for the remaining variance. The fact that differences in body composition do not totally explain variations in maintenance needs is supported by Taylor et al. [121], who observed persistent differences between beef and dairy cattle when animals were evaluated at identical body composition.

#### 4.2.4. Physical Activity

Luiting et al. [122] found that physical activity was the most influential factor in determining energy efficiency, accounting for 80% of the heritable variance in RFI. Morrison and Leeson (1978), as cited in Archer [38], discovered that productive birds were less active (spent more time resting and less time upright and consuming) compared to less productive birds. Similarly, Katle and Kolstad [123] discovered that locomotive activity was the most significant factor influencing FE. There have been few attempts to replicate these results with larger ruminant species. Herd and Arthur [124] discovered that DMI variation in cattle may be associated with disparities in activity levels, including feeding, ruminating, and walking at different speeds. Both Herd et al. [125] and Richardson [126] discovered that physical activity accounted for 5–10% of the variance in DMI. Physical activity may influence total energy expenditure; if an increase in DMI does not compensate for the energy consumed, overall gain and efficiency may be diminished [124,125,127]. According to Llonch et al. [128], animals that walked less had a greater DMI.

#### 4.2.5. Extra-Physiological Considerations

The efficiency with which feed is utilized for maintenance and production may also be influenced by a vast array of biological systems within the animal. Dry matter digestibility measures an animal’s ability to derive usable nutrients from its dietary source. According to research by Richardson et al. [129], there was a small but significant difference in digestibility between cattle with high and low RFI. Herd et al. [130] observed that ewes from a line selected for high weaning weight assimilated 1.8% more dietary organic matter than ewes from a line selected for low weaning weight. This suggests that digestibility is related to genetic variations in performance. According to Katle and Kolstad [123], the digestibility findings from their investigation into the causes of variation in hens’ residual feed consumption were ambiguous.

Numerous other physiological indicators have been linked to performance in different studies. Richardson et al. [129] discovered that cattle with a high RFI (i.e., low efficiency) had a higher total plasma protein concentration than cattle with a low RFI (i.e., high efficiency). These differences may have resulted from distinct metabolic processes, such as protein synthesis and proteolysis rates, and not the immune system. Müller et al. [38] demonstrated that milk lipid, milk protein, thyroxine (T4), triiodothyronine (T3), T3/T4, and total plasma protein correlate with dairy cattle’s residual feed consumption. These findings suggest that animal-to-animal differences in efficiency are associated with differences in metabolism. However, the physiological mechanisms underlying this variation and the relative importance of various metabolic processes in determining FE are still unknown. This information is necessary for a deeper understanding of the processes underlying variations in supply efficiency [38].

#### 4.2.6. Visceral Organs

Differences in metabolic activity between lean and fat tissue help to clarify variations in maintenance requirements. Recent research has concentrated on other highly metabolic body tissues. Smith and Baldwin [131] demonstrated that the liver, heart, breast, and gastrointestinal tissues are among the most metabolically active. They hypothesized that alterations in the relative proportions of these tissues and organs contribute to the increase in maintenance requirements of lactating dairy cows. Similarly, Early et al. [132] observed that the turnover of proteins in visceral tissues was greater than that of skeletal muscle. According to research by Ferrell and Jenkins (1985), as cited in Archer et al. [38], visceral organs consume most of the nutrients required for basal metabolism. The high consumption of these tissues was attributed to their high protein synthesis rates. Consequently, the proportion of these visceral organs within the body is anticipated to impact the maintenance requirements of cattle.

Jenkins et al. [133] found that the proportion of non-carcass components in dairy-type heifers was higher than in beef cattle. In the study by Jenkins et al., Brown Swiss cows had substantially larger livers and lungs than Hereford cows, with significantly smaller internal organs and more structural-type tissue. The effect of visceral organs on total body nutrient consumption is demonstrated by the response of these organs to dietary manipulations. The increased weights or proportions of the small intestine, liver, and pancreas [134], and the small and large intestines, liver, and stomach [38,133,135], have been associated with the increased energy demands observed in studies of sheep and cattle fed high levels of nutrition. Some research suggests that restricting nutrition to maintain a live weight increases the proportion of metabolically inactive viscera, such as the liver and digestive tract [136]; however, others have found the opposite to be true [137].

#### 4.2.7. Intestinal Absorption and Cell Morphology

Increased intestinal nutrient absorption has been linked to variations in FE among animals [114]. This is supported by the negative correlations (r = −0.33) between jejunal mucosal density and RFI in cattle [138]. Montanholi et al. [139] found more cells in the duodenum and ileal epithelial tissue of low-RFI steers compared to high-RFI steers of the same age. In a 2013 study, single nucleotide polymorphisms (SNPs) in genes responsible for transporting phospholipids and cholesterol in the small intestine were associated with disparities in FE [140].

Improving FE offers a direct pathway to reducing the environmental footprint of beef production. This aligns with global sustainability priorities such as those outlined in the United Nations Sustainable Development Goals [141]. In summary, feed-efficient cattle require fewer resources to produce the same output, reducing the demand for feed crops, minimizing land use, and lowering greenhouse gas emissions associated with feed production and digestion [142,143]. By targeting traits such as RFI in genetic and genomic selection, producers can identify and propagate animals that convert feed into body mass more effectively, thereby reducing the beef sector’s contribution to environmental degradation. These efforts resonate with global calls for sustainable agricultural intensification, which aim to balance productivity with environmental conservation. Emphasizing the role of FE in mitigating climate change and preserving natural ecosystems not only enhances the relevance of these approaches but also underscores their potential to meet international climate targets [144,145]. By reducing environmental impact through FE, the beef industry can demonstrate leadership in addressing critical global challenges while ensuring long-term sustainability.

The relationship between genetic/genomic selection for FE and environmental sustainability requires careful consideration of potential trade-offs and uncertainties. While reducing FI through genetic selection can lower overall resource requirements and emissions per unit of output, this outcome is not guaranteed across all production scenarios. For example, larger cows with improved FE may demand greater energy intake for maintenance, which could offset some of the anticipated environmental benefits of reduced FI. Additionally, life-cycle assessments are necessary to account for the broader impacts, including methane emissions and nutrient cycling, to avoid oversimplifying environmental outcomes [18,146]. Such assessments ensure that the ecological trade-offs inherent in selection programs are accurately represented and addressed.

Moreover, genetic selection for feed efficiency could inadvertently result in rebound effects, where improvements in FE enable increased production scale, amplifying total resource use and environmental impact. These potential unintended consequences highlight the need for an integrative approach to sustainability that considers genetic, nutritional, and management factors holistically across the production cycle [145]. This approach would involve ongoing monitoring of environmental outcomes and adaptive management practices to mitigate adverse effects while capitalizing on the benefits of selection strategies [147]. Future research should aim to develop more nuanced strategies that balance feed efficiency improvements with sustainable resource use, ensuring that the genetic advancements contribute positively to environmental and production goals.

## 5. Nutritional Models

### 5.1. Descriptors of International Nutritional Models on Determination of Energy Requirements for Beef Cattle

Table 3 summarizes the nutritional models used worldwide to estimate beef cattle’s calorie requirements.

In contrast to the comparative slaughter trials utilized by the North American [148,149] and Brazilian systems [150], the AFRC, CSIRO [151], and INRA [152] models are based on calorimetry. The energy systems that were initially developed using calorimetric data for dairy cattle in the United States may also be beneficial for heifer calves [153]. However, the NRC notes that calorimetry estimates have limitations when applied to realistic feeding conditions [154]. To supplant the defective Starch Equivalent (SE) method, British researchers proposed in 1965 a calorimetric nutrition system based on metabolizable energy (ME) [155]. Under The Agricultural and Food Research Council’s vigilant eye, the Metabolizable Energy (ME) system, ARC, 1980 as cited in [17], underwent simplification [156], revision, and enhancement [157]. An energy and protein requirements guidebook for ruminants was published in the early 1990s [158].

Calorimetry is a technique for calculating ME by measuring heat emission and subtracting losses from total energy consumption. Indirect calorimetry, which calculates heat production from oxygen intake, carbon dioxide production, and nitrogen excretion, is more accurate than comparative slaughter [159]. Maintenance energy (ME_m_) is the disparity between the quantity of heat produced during a fast and the amount of sustenance consumed during the fast. When attempting to estimate energy balance and other variables, such as diet, output, organ mass, breed, sex, and length arise [159,160].

ME_m_ can be determined by either dividing the fasting metabolic rate by the energetic efficiency for maintenance (K_m_) or by regressing energy intake against energy outputs. The ME method has faults due to its foundation in experiments with castrated male sheep rather than cattle, even though there are no significant differences in energy consumption between the two species [161]. Since more than a century ago, calorimetry investigations have provided the foundation for our understanding of energy metabolism in domesticated animals, despite being costly and time-consuming [162]. The current Australian method for calculating the energy requirements of beef cattle is based on the UK system’s definitions of animal energetics. However, the AFRC’s energy recommendations are no longer applicable due to the constant evolution of the dairy and cattle industries [158]. As part of an endeavor to modernize the dietary requirements of dairy cattle, the ‘Feed into Milk’ technique was devised in 2004 [163]. The energy requirements of beef cows are calculated using the California Net Energy System and slaughter comparisons. It is more convenient to discuss energy requirements in terms of either Shrunk Body Weight (SBW) or empty body weight (EBW), although live weight (LW) is the most significant factor in [164] determining maintenance demands.

Directly quantifying ME and retained energy (RE) at slaughter for comparison enables linear regression to estimate ME consumption for RE. This method may also be used to calculate the fasting heat production (FHP) and maintenance energy (ME_m_). However, comparative slaughter research is time-consuming, labor-intensive, and costly. Cattle weighing less than 250 kg do not reliably suit the NASEM energy calculations. Long-term feeding experiments can estimate maintenance requirements by calculating the quantity of food required to maintain a constant body mass [148]. However, when information is rare in the literature on several factors, custom-tailored production studies may be helpful in modifying energy requirements. The feed into the milk system for dairy cattle is one example of such studies, effectively incorporating custom-tailored production into updated standards [163]. The ME and NE systems include maintenance, production (milk, LW gain, fiber growth), and fetal development when calculating the energy requirements of ruminant animals [159]. ME [151,158] and NE [148,150,152] systems both utilize feed ME concentration in their calculations; however, the NE system also accounts for variable NE values based on animal production functions.

Due to concerns regarding the cattle industry’s impact on global warming, NE [148,150,152] systems now include equations to predict enteric methane output. Using their own sets of equations, both the tropical and French methodologies consider individual cattle genotypes [152]. Concerns have been expressed regarding the energy requirements of foraging and cold-stressed animals. These systems use a factorial design, which has been criticized for its inability to describe the interactions between feed and nutrients adequately. This analysis excludes the more mechanical Ruminant Nutrition System (RNS) [149]. It is recognized that the RNS’s theoretical foundation is crucial to its future development. There are numerous European feeding systems, with the French system being the most recently enhanced. Comparatively, the Brazilian method was designed for tropical regions due to its importance to beef cattle energetics, particularly from 2009 to 2020.

### 5.2. Metabolizable and Net Energy Requirements for Maintenance for Growing Beef Cattle from Recent Studies Published Around the World

According to Ferrell and Jenkins [165], up to 65–70% of the energy required for livestock production is spent on maintenance. To optimize the utilization of dietary energy, it is essential to determine the maintenance energy requirements with precision. Maintenance energy requirements vary based on variables such as live weight, metabolic body size, age, breed, sex, and production level [165,166]. Diverse models are developed using the live weight of animals, either as metabolic live weight (LW^0.75^) in calorimetry-based systems [151,152,158] or adjusted to empty body weight (EBW) in comparative slaughter-based models [148,150]. Notably, the AFRC equation raises LW to the power of 0.67, whereas the CSIRO and INRA equations both use a coefficient of 0.75. The AFRC equation isolates maintenance energy requirements into fasting metabolism and adds energy cost for activity, whereas the CSIRO equation incorporates the factor (0.1 ME_p_ × k_m_) into the basal metabolic rate to account for increased maintenance requirements with higher FI. Table 4 presents the equation used to estimate maintenance requirements in animals.

However, Marcondes et al. [167], utilizing an earlier BR-corte database, did not detect a distinct relationship between k_m_ and ME concentration in the diets of tropical animals fed low-digestibility feeds. Both the AFRC and CSIRO systems incorporate a correction factor of 1.15 to account for the elevated metabolic rate of bulls compared to steers and heifers.

The Australian methodology accounts for breed differences by employing correction factors of 1.2 for *Bos taurus indicus* and 1.4 for *Bos taurus taurus*. The effect of age is included as a power term in the CSIRO equation. In NE systems, it is presumed that the maintenance energy requirement is constant per kg of LW^0.75^ (Table 4). The French system differentiates between pre-ruminant and ruminant animals using distinct coefficients (0.289 and 0.423, respectively). The NASEM system additionally compensates for the impact of environmental temperature on metabolic rate, assuming thermoneutrality at 20 °C and considering cold or heat stress. Live weight (LW) and maintenance energy (ME_m_) for *Bos taurus* bulls were obtained using equations from the three energy systems above. For all calculations utilizing the NASEM equation, thermoneutrality was assumed. Because of the following, this comparison of energy systems does not include the CSIRO or BR-Corte equations: The Brazilian equation was derived from data from Zebu and Zebu crossbred animals, which is not representative of UK conditions. The CSIRO equation requires growth curve data and implies non-constant ME for production even at the same q value. The BR-Corte approach provides an equation only for *Bos taurus taurus × Bos taurus indicus* crossbreeds and not for pure *Bos taurus taurus* animals, which is another disadvantage. The NRC estimates that the net energy required for maintenance (NE_m_) of *Bos indicus* cattle is approximately 10% less than that of *Bos taurus* cattle. With increasing metabolic weight (LW^0.75^), the NE_m_ per kg requirement in all three energy systems decreases. The ME_m_ from the INRA equation (ruminant equation) was frequently greater than the ME_m_ from the AFRC and NASEM equations. For LW less than 180 kg (pre-weaning), however, the associations between the two European systems were comparable. Intriguingly, the results of the INRA equation for pre-ruminant animals are remarkably similar to those of the NASEM equation, albeit with substantially lower ME_m_ values. Using *Bos taurus* genotypes, AFBI calorimetry researchers determined that developing animals require 21% more ME_m_ than mature animals (0.78 vs. 0.617 MJ/kg LW^0.75^). Table 5 demonstrates that this is consistent with the findings of [166,168], as well as the comparisons conducted by Jiao et al. [169].

Various physiological states influence the requirement for NE_m_ in developing animals, as demonstrated by comparative euthanasia experiments conducted in Brazil. At 0.334 ± 0.0335 MJ of NE_m_/kg LW^0.75^ versus 0.349 ± 0.0420 MJ of NE_m_/kg LW^0.75^ (finishing animals versus developing animals, respectively; *p* = 0.426), the NE_m_ demand of bulls weighing 300 kg or less was 4.3% lower. This evaluation did not include additional comparisons based on measuring method, breed, or sex due to a lack of data.

The net energy for maintenance (NE_m_) requirements in the earlier data compiled by Cottrill et al. [182] were slightly higher (0.353 vs. 0.336 MJ/kg LW^0.75^, respectively; see Table 5) compared to the most recent data, which consisted primarily of comparative slaughter trials with Zebu animals and their crossbreeds. Variations in the conversion of metabolizable energy (ME) to net energy (NE_m_) for maintenance at the level of individual animals and in the method for calculating k_m_ may contribute to the observed differences in ME_m_ levels between studies. The estimated ME_m_ requirement using the AFRC and NASEM equations appears to be 8.2% and 19.5% less than the mean value of 0.672 MJ/kg^0.75^ derived from calorimetry studies conducted at AFBI (Table 5) based on a hypothetical *Bos taurus* bull with a live weight (LW) of 300 kg and a constant km value of 0.65. In accordance with the data, the INRA calculation for ruminant animals predicts a value of 0.67 MJ/kg^0.75^ (see Table 4 for additional information). Cottrill et al. [182] discovered that the ME systems recommend a 1.15-fold higher ME_m_ maintenance requirement for males than for steers and heifers (Table 4). These findings are supported by the results of Jiao et al. [169].

Selection based on RFI has been extensively studied worldwide over the past decade, and its effects on the maintenance energy requirements of developing animals in Irish settings have been partially elucidated. Lawrence et al. [183] determined NE_m_ requirements for developing Simmental Holstein-Friesian heifers categorized according to phenotypic RFI under Irish conditions based on a regression analysis of daily live weight gain (g/kg LW^0.75^) versus NE intake. The high RFI group required 0.410 MJ/kg LW^0.75^, the medium RFI group required 0.368 MJ/kg LW^0.75^, and the low RFI group required 0.335 MJ/kg LW^0.75^ (LW = 311 kg at the beginning of the test period).

Cabezas-Garcia et al. [17] found that animals with a high RFI required 18% more ME_m_ than those with a low RFI (0.777 versus 0.637 MJ of ME_m_/kg LW^0.75^ for the high and low RFI groups, respectively). These values are consistent with those obtained by Gomes et al. [180] in Nellore calves (Table 5). The NE_m_ values derived by Lawrence et al. [183] were omitted from Table 5 as energy metabolism calculations were not the primary focus of the investigation.

Energy systems provide equations for estimating net energy requirements for weight gain (NE_g_) in developing cattle, as shown in Table 6.

Different systems utilize BW and growth data to estimate nutritional energy requirements, with specific methods incorporating unique adjustments for accuracy. The LW and LWG are incorporated into the calculations and are utilized in numerous systems, including AFRC, CSIRO, NASEM, and BR-Corte. The AFRC equation adjusts the NE_g_ to account for differences in maturation between sexes of the same breed, with greater correction factors for the former. The French method incorporates protein and lipid retention to improve NE_g_ estimates when comparing systems based solely on LW measurements.

Previously, it was believed that there were substantial differences in the fasting or maintenance energy expenditure of cattle due to differences in body composition. It is now possible to obtain precise and consistent readings using non-invasive techniques such as computed tomography and ultrasound. Concerns persist, however, about the viability of employing such techniques on farms. The projected energy required for LW gain is based on RE about the animal’s maturation, which in turn is primarily determined by the composition of EBG. According to studies, animals’ maintenance energy requirements decrease by 0.75 kcal/kg of LW as they acquire weight. This trend may be explained by the decreasing relative weight of organs and body protein that occurs with aging. The American method for calculating NE requirements for growth considers current and desired BW. For heifers and bulls, it is suggested that various variables be used to calculate the net energy requirements for weight gain.

Males have greater growth potential on a diet rich in forage than heifers. Regarding lean growth, males respond more significantly to increases in FI per MJ of metabolizable energy (ME) than females. A developing animal’s total energy requirements are believed to include maintenance and growth requirements. Comparing the AFRC and NASEM energy requirements using a hypothetical example from Gordon et al. [168]. The minimal energy requirement (ME_min_) calculated by both systems is significantly below the actual value. Both methods overestimate the efficiency with which ME is converted to growth (in kilograms). The formulas used to determine a meal’s metabolizability may influence efficiency estimations, particularly for k_g,_ as shown in Table 7.

As addressed by the French method, muscle lipid and protein accretion rates could improve NE_g_ calculations for developing animals in Northern Ireland’s climate. Modernizing the British approach requires a reevaluation of how the concept of food metabolizable energy influences energy utilization efficiency.

When animals experience a period of malnutrition followed by adequate food intake, they experience compensatory growth, which increases the efficiency with which they use energy to gain weight. Beef cattle raised on pasture, where forage quantity and quality fluctuate with the seasons, provide an intriguing case study for this issue. When determining an animal’s energy requirements at a younger age, the British system disregards compensatory growth, whereas the Australian system considers it. While the most recent upgrade to the French system acknowledges the significance of compensatory growth, it does not factor it into estimates of energy requirements. According to the National Research Council (NRC), animals experiencing compensatory growth have more energy available for weight gain because their maintenance requirements are reduced. Compensating animals have become more efficient at utilizing energy, requiring less net energy to gain mass. Compensatory growth has been linked to factors such as gut content, increased tissue intestine weight, and internal organ size alterations. However, compensatory growth in animals occurs as a direct result of two factors. First, cattle experiencing compensatory growth are leaner than average, which leads to a lighter weight due to reduced muscle and fat, while their frame and gut capacity remain normal. As a result, these cattle can consume more feed than their live weight alone would predict. Second, because they are lean, their weight gain is more efficient per unit of energy intake, as the gain is primarily lean tissue (protein and water) rather than fat. This is important because the energetic cost of depositing fat is much greater than that of lean tissue. Age may modify the magnitude of compensatory growth responses. It is still being determined how compensatory growth affects the caloric requirements of beef cattle, particularly in the United Kingdom and Ireland.

### 5.3. Energy Requirement for Maintenance During the Finishing Period

Multiple studies have demonstrated a variance in the maintenance energy requirements of various beef cattle breeds. However, there is limited information on studies comparing the energy requirements of different genetic groups during the finishing period. One of the studies that attempts to bridge this knowledge gap is that of Goulart et al. [184]. In the study, animals were fed the same diet from birth to slaughter to compare the NE_m_ of purebred Nellore to that of its crosses with Simmental, Angus, and Canchim breeds. The animals were born in the tropics and fed free-choice minerals throughout their development until the feedlot phase began, potentially affecting factors such as ingestion, carcass composition, mature weight, and the ensuing energy requirement for maintenance during the finishing phase. The dietary digestible energy (DE) was determined to be 4409 Mcal/kg of total digestible nutrients. As recommended by NASEM [148], they utilized an efficiency of 82% to convert DE to ME for both the limit-fed and ad libitum groups. Recent publications suggest that the ratio of DE to ME may exceed 82% and can differ [185,186]; however, Goulart et al. [184] chose to use the established 82% ratio. This decision was based on the high proportion of forage in their diets, which is comparable to the diets used in previous studies that determined the 82% ratio [149]. The Lofgreen and Garrett [164] method calculated the RE and maintenance energy requirements. The initial EBW was calculated using SBW. Then, the initial empty body fat (EBF) and empty body protein (EBP) were estimated for each animal and genetic group (GG) using the average EBW, SBW, EBF, and EBP values of the respective breed group’s baseline cattle. The methodology Goulart et al. [184] used is comparable to that described by Tedeschi et al. [187].

Goulart et al. [184] found no significant differences in NE_m_ requirements between Nellore (NL) and Angus (AN) crosses at the same age and frame size (*p* = 0.528), Charolais (CN) crosses (*p* = 0.671), and Simmental (SN) crosses (*p* = 0.706). Furthermore, they determined a common NE_m_ requirement of 86.86 kcal/d/kg^0.75^ EBW (see Table 8) by analyzing the aggregated data for NL, CN, AN, and SN. This equates to 79.6 kcal/d/kg^0.75^ EBW for a 300 kg SBW bull, which is very similar to the value of 77 kcal/d/kg^0.75^ EBW reported by Lofgreen and Garrett [164]. Fox and Black [188] also noted that beef cattle strains with comparable frame proportions are presumed to have comparable net energy needs at the same body composition. According to the NRC [154], it was assumed that *Bos taurus indicus* require 10% less NE_m_ than *Bos taurus taurus*, with crossbreeds falling in the middle.

Chizzotti et al. [189] observed a 14% reduced NE_m_ requirement for Nellore purebreds and crossbreds, with an average NE_m_ of 75 kcal/d/kg^0.75^ EBW, in contrast to the findings of Goulart et al. [184] (86.8 kcal/d/kg^0.75^ EBW). In their meta-analysis, Marcondes et al. [176] reported a NE_m_ of 79.4 kcal/d/kg^0.75^ EBW for Nellore cattle. Tedeschi and Fox [190] reported a 95% confidence interval of 74.1 to 84.7 kcal/d/kg^0.75^ EBW for Nellore cattle, and the average NE_m_ of 77 kcal/d/kg^0.75^ EBW reported by Lofgreen et al. [164] would lie within the range of meta-analysis databases. Frisch and Vercoe [191] suggested that Zebu cattle require 10% less NE_m_, which is consistent with the findings of Goulart et al. [184]. In contrast, the experimental findings of Ferrell and Jenkins [192] and Tedeschi et al. [187] do not support the notion that Nellore cattle have a diminished NE_m_. The reported NE_m_ value of 86.8 kcal/d/kg^0.75^ EBW is comparable to the NE_m_ reported by Ferrell and Jenkins [192] for Brahman crossbred steers and Tedeschi et al. [187] for Nellore steers. The recent publication of NASEM [148] also noted that *Bos indicus* cattle, specifically those reared in Brazil, do not require a 10% NE_m_ adjustment. Despite the lack of statistical significance, the study revealed that the NE_m_ values for NL and AN were 85.53 and 90.76 kcal/d/kg^0.75^ EBW, respectively, indicating a 5.76% decrease in NE_m_ for NL compared to AN. This relative difference between purebred NL and AN crossbred calves corresponds with NRC [154] data. Oldham [149] mentioned the concept of reduced NE_m_ requirement while maintaining similar basal metabolism. Because breed genetics and nutritional management practices change over time, more effort should be devoted to studying and comprehending the NE_m_ of cattle. Comparisons of maintenance requirements should also consider the genetic background of the tested population. Moraes et al. [193] found that selection for growth considerably increased the maintenance requirements of Nellore and Holstein cattle over time.

Moraes et al. [193] assumed that the ME_m_ requirement for different genetic groups was similar, with a mean value of 137.53 kcal/d/kg^0.75^ EBW. This is because there were no significant differences between GG for the slope and intercept when regressing heat production on metabolizable energy intake. The average values for k_m_ and k_g_ among GG were also comparable (*p* > 0.05 for both): 63.2% and 26.0%, respectively. Ferrell and Jenkins [192] reported similar k_g_ and k_m_ values (between 65% and 69%) for Brahman crossbred steers, whereas Oldham [149] reported higher k_g_ and k_m_ values (69.9% and 52.5%, respectively) for Nellore steers. Chizzotti et al. [194] reported that the k_m_ and k_g_ values for Nellore Red Angus crossbred cattle were 70.6% and 47.0%, respectively.

To calculate NE_m_ based on comparative slaughter methods, animals must be fed at two or three levels of ingestion (approximating maintenance, libitum, and an intermediate level) [164]. Variations in RE and body mass index (BMI) resulted from these dietary differences. Goulart et al. [184] were among the first to compare the energy needs of various GG raised under the same conditions and fed the same diet throughout their lives. In their study, cattle were assigned to two nutritional treatments (NT): ad libitum or restricted feeding. As corroborated by previous studies by Old and Garrett [195] and Oldham [149], the limit-fed treatment received 70% of the daily feed administered to the ad libitum treatment of the same genetic group. However, it is essential to observe that increased FI is a significant component of compensatory growth when animals are fed at total capacity [148]. In their study, ad libitum-fed steers had a higher average daily DMI as a percentage of BW than limit-fed steers (2.90% of BW in ad libitum-fed steers, compared to 2.10% of BW in limit-fed steers). They suggested that the ad libitum-fed group experienced compensatory growth, although if cattle consume more ad libitum, assuming the same diet is fed, the increased intake of energy and protein should lead to increased weight gain due to the availability of additional nutrients for growth. Furthermore, higher FI would result in a dilution of the energy cost for maintenance, directing more nutrients toward weight gain. This raises questions about whether the observed growth in the ad libitum-fed group is truly compensatory or simply a result of higher nutrient intake. The higher values of DMI were reportedly linked to lower quality and quantity of pastures because the animals experienced a protracted period of feed restriction, which resulted in decreased growth rates, particularly from 18 months of age until the commencement of the feedlot phase. According to Lofgreen and Kiesling [196], compensatory growth periods are typically characterized by increased FI throughout the feeding period. Consequently, limiting the limit-fed treatment to 70% of the daily feed offered to the ad libitum treatment likely overestimated the feed offered to the limit-fed cattle, thereby influencing the net energy requirement calculations in their study. As a result, their data represents the first study to demonstrate how the energy requirements of cattle can change when animals experience compensatory gain.

## 6. Conclusions

The beef industry faces increasing pressure to enhance FE while minimizing environmental impacts and maintaining productivity. Current strategies to improve FE often involve trade-offs, such as potential reductions in growth, carcass quality, or reproductive performance. Achieving significant advancements in FE without compromising overall productivity remains a critical goal. In this context, genetic selection for FE traits presents a promising yet challenging avenue for future research and development. Historically, the high cost and complexity of measuring FE have constrained its routine application in breeding programs. However, recent advancements in technology, such as automated FI recorders, precision sensors, and machine learning algorithms, are transforming the landscape of phenotyping. Machine learning approaches can predict FE phenotypes using readily available data, including growth rate, body condition, and behavioral traits, significantly increasing the number of usable phenotypes. As these advanced tools are adopted across farms and feedlots, the resulting datasets can support the development of more robust and accurate genomic selection models. With sufficient phenotypic data collected across diverse production environments, genomic models will enable breeders to make more precise selection decisions.

## 7. Future Directions

The developing area of nutrigenomics, the study of how diet can change and/or modify gene expression, offers a possible avenue for improving FE. By accounting for genetic variation among animals, nutrigenomics could optimize FE at the individual level, i.e., precision nutrition. This approach could allow producers to adjust diets based on each animal’s genetic profile, thereby improving feed conversion efficiency to meet specific nutritional needs and production targets. Another future-focused approach that warrants further investigation is the incorporation of nutritional models into genetic evaluations for FE. Nutritional models provide valuable insights into how different feeding strategies affect growth and metabolism and, thus, provide a way of accounting for environmental and dietary variations when predicting FE traits. By integrating these models, which could be incorporated into genetic evaluations, we can achieve more accurate estimations of an animal’s genetic potential and, consequently, enable the development of feed-efficient cattle across a range of production systems and feed resources. Additionally, there is a need for better equations to predict DMI, particularly for cattle on high-forage diets. The variation in forage quality, particle size (e.g., long vs. chopped), levels of supplemental feeds, and the amount of feed offered relative to animal capacity (ad libitum vs. limit-fed) all significantly influence intake and the digestibility of the total diet. In feedlot finishing diets, although there is animal-to-animal variation in intake, diet quality plays a much smaller role as a source of variation. Addressing these gaps in intake prediction is essential for improving the precision of nutritional models and enhancing their utility in genetic evaluations.

## Figures and Tables

**Table 1:** Heritability estimates (SE in parentheses) for average daily gain (ADG), body weight (BW), feed intake (FI), residual feed intake (RFI), and feed conversion ratio (FCR) for a range of studies in growing animals across different breeds and countries ^1^.

ADG	BW	FI	RFI	FCR	Breeds ^2^	Country	Animals ^3^	Reference
0.65 (0.13)	-	0.64 (0.12)	0.28 (0.11)	-	AN, HE, SH	United States	1324	[34]
0.36 (0.11)	-	-	-	0.14 (0.07)	AN	United States	393	[66]
0.33(0.11)	-	-	-	0.13 (0.08)	HE	United States	340	[66]
-	-	-	-	0.33 (0.10)	HE	United Kingdom	452	[44]
0.48 (0.21)	0.39 (0.19)	0.37 (0.19)	-	0.19 (0.16)	Bonsmara	South Africa	298	[67]
0.48 (0.21)	-	0.06 (0.12)	-	0.46 (0.20)	FRXHE	United Kingdom	327	[68]
0.43 (0.24)	0.45 (0.22)	0.27 (0.15)	0.23 (0.12)	0.35 (0.22)	AN	Canada	263	[69]
0.16 (0.15)	0.43 (0.22)	0.18 (0.10)	0.07 (0.13)	0.08 (0.09)	HE	Canada	271	[69]
0.55 (na)	0.51 (na)	0.58 (na)	-	0.16 (na)	BB	France	1442	[70]
0.25 (na)	-	0.24 (na)	-	0.14 (na)	HE	United States	486	[71]
0.35 (0.11)	-	0.62 (0.12)	0.62 (0.14)	0.42 (0.13)	AN, HE, Polled HE, SH	Australia	760	[72]
0.41 (0.08)	0.68 (0.08)	0.59 (0.07)	0.44 (0.07)	0.31 (0.09)	AN, HE, SH	Australia	966	[72]
0.38 (0.10)	0.42 (0.10)	0.31 (0.08)	0.16 (0.08)	0.17 (0.09)	HE	United Kingdom	540	[62]
0.28 (0.04)	0.40 (0.02)	0.39 (0.03)	0.39 (0.03)	0.29 (0.04)	AN	Australia	1180	[5]
0.34 (0.04)	0.37 (0.04)	0.48 (0.04)	0.39 (0.04)	0.46 (0.04)	CH	France	792	[57]
0.41 (0.06)	0.46 (0.05)	0.48 (0.06)	0.43 (0.04)	0.31 (0.06)	CH	France	397	[57]
-	-	-	0.30 (0.06)	-	CH-sired steers	Canada	281	[73]
-	-	-	0.26 (0.07)	-	CH-sired steers	Canada	274	[73]
0.23 (0.06)	0.41 (0.07)	0.27 (0.06)	0.18 (0.06)	0.06 (0.04)	Tropically adapted, temperate	Australia	1481	[63]
0.35 (0.03)	0.35 (0.02)	0.44 (0.06)	0.38 (0.07)	0.37 (0.06)	CH, LI, AN, SI, HE, BA	Canada	2284	[74]
0.37 (na)	-	-	0.31 (na)	0.34 (na)	Bonsmara	South Africa	6738	[75]
0.20 (0.10)	0.47 (0.10)	0.34 (0.11)	0.24 (0.11)	0.15 (0.04)	Japanese Black (Wagyu)	Japan	740	[76]
0.59 (0.17)	0.32 (0.14)	0.54 (0.15)	0.21 (0.12)	0.41 (0.15)	AN, CH, composite	Canada	464	[77]
0.26 (na)	0.39 (na)	0.33 (na)	0.29 (na)	0.14 (na)	Wagyu	Japan	1304	[78]
-	-	0.36 (0.09)	0.49 (0.09)	0.38 (0.07)	Wagyu	Japan	514	[79]
0.34 (0.12)	0.47 (0.16)	0.49 (0.15)	0.24 (0.11)	-	Brahman	Australia	1007	[80]
0.20 (0.10)	0.39 (0.13)	0.51 (0.14)	0.38 (0.12)	-	Tropical Composite	Australia	1209	[80]
0.21 (0.12)	0.35 (0.15)	0.48 (0.14)	0.47 (0.13)	0.29 (0.12)	Brangus	United States	468	[81]
-	-	-	0.18 (0.14)	-	AN, CH, composite	Canada	387	[82]
0.09 (na)		0.14 (na)	0.14 (na)	-	AN	United States	698	[83]
-	0.57 (0.10)	0.30 (0.08)	0.26 (0.10)	0.30 (0.12)	BA	France	678	[84]
-	0.30 (0.08)	0.48 (0.14)	0.45 (0.18)	0.23 (0.15)	LI	France	708	[84]
0.30 (0.06)	0.69 (0.07)	0.49 (0.07)	0.45 (0.07)	0.30 (0.06)	AN, CH, HE, SI, LI	Ireland	2605	[28]
-	-	0.21 (0.07)	0.14 (0.06)	0.18 (0.07)	AN, BR, BA	United States	1129	[85]
0.06 (0.08)	-	0.30 (0.15)	0.19 (0.12)	0.07 (0.09)	ANX, CHX	Canada	402	[86]
0.17 (0.28)	-	0.43 (0.14)	0.36 (0.13)	0.26 (0.12)	ANX, CHX	Canada	419	[86]
-	-	0.70 (0.11)	0.22 (0.07)	0.11 (0.05)	Wagyu	Japan	863	[87]
0.26 (0.04)	0.33 (0.03)	0.36 (0.05)	-	-	AN	United States	4215 to 18,169	[88]
0.28 (0.11)	-	0.41 (0.12)	0.29 (0.12)	-	AN, CH, composite	Canada	721	[25]
0.26 (0.10)	0.35 (0.12)	0.40 (0.02)	0.52 (0.14)	0.27 (0.10)	Multibreed	United States	1141	[89]
0.30 (0.06)	0.69 (0.07)	-	-	0.30 (0.06)	AN, CH, HE, LI, SI	Ireland	3531	[90]
0.38 (0.18)	-	-	0.27 (0.12)	-	-	France	2023	[91]
0.38 (0.12)	-	-	0.47 (0.12)	0.21 (0.08)	AN, CH	Canada	968	[92]
-	-	-	0.40 (0.10)	-	AN, ANXSI, SI	United States	1321	[93]
0.35 (0.15)	-	-	0.38 (0.16)	0.31 (11)	NE	Brazil	1038	[50]
0.20 (0.03)	-	-	-	-	AN, HE, MARC III, SI, LI, CH, RA	United States	6331	[94]
0.33 (0.07)	-	0.55 (0.08)	0.40 (0.07)	0.20 (0.06)	AN	Australia	6371	[47]
0.53 (0.12)	-	-	0.25 (0.11)	-	AN, HE, MARC III, SI, LI, CH, RA	United States	687	[65]

^1^ na = not available. ^2^ AN = Angus; BA = Brangus; BB = Belgian Blue; BR = Brahman; CH = Charolais; CHX = Charolais crossbred; FRXHE = Friesian-Hereford crossbreds; HE = Hereford; LI = Limousin; SH = Shorthorn; SI = Simmental; RA = Red Angus; GE = Gelbvieh, NE = Nellore; MARC III = Composite breed (¼ Angus, ¼ Hereford, ¼ Pinzgauer, ¼ Red Poll). ^3^ Number of animals.

**Table 2:** Heritability estimates (SE in parentheses) for body weight (BW), feed intake (FI), residual feed intake (RFI), and feed conversion ratio (FCR) for a range of studies in mature animals across different breeds and countries ^1^.

BW	FI	RFI	FCR	Breeds ^2^	Country	Animals	Reference
0.65 (0.01)	-	0.04 (0.05)	-	Norwegian	Norway	353	[39]
0.29 (0.09)	0.02 (0.02)	0.23 (0.11)	0.18 (0.15)	HE	Canada	295	[66]
0.40 (0.04)	0.11 (0.02)	0.03 (0.01)	0.11 (0.06)	HE, multibreed	Canada	1174	[66]
0.20 (0.12)	0.03 (0.01)	0.03 (0.02)	0.11 (0.10)	HE	Canada	206	[95]
0.44 (0.17)	0.16 (0.02)	0.22 (0.04)	0.05 (0.01)	HE, multibreed	Canada	729	[95]
0.71 (na ^1^)	0.28 (na)	0.23 (na)	0.26 (na)	AN, HE, Polled HE, SH	Australia	751	[54]
-	-	0.16 (0.10)	-	AN, HE, MARC III, SI, LI, CH, RA	United States	622	[65]

^1^ na = not available. ^2^ AN = Angus; HE = Hereford; SH = Shorthorn; SI = Simmental; LI = Limousin; CH = Charolais; RA = Red Angus; MARC III = Composite breed (¼ Angus, ¼ Hereford, ¼ Pinzgauer, ¼ Red Poll).

**Table 3:** General descriptors of international nutritional models on the determination of energy requirements for beef cattle.

Country	Organization	Date	Breed	Maintenance Requirement/Units	Observations
UK	Agriculture and Food Research Council, AFRC, formerly Agriculture Research Council (ARC)	1993	Continental and British breeds	Calorimetry/ ME	Continues to offer a crucial theoretical foundation for the majority of energy systems worldwide. Forage-based diets.
Australia	Australia Commonwealth Scientific and Industrial Research Organization (CSIRO)	2007	*Bos t. taurus*, *Bos t. indicus*, and crossbreds.	Calorimetry/ ME	The CSIRO guidelines align with the AFRC approach, utilizing ME_m_ to measure maintenance requirements. The feed tables also incorporate low-quality forages.
France	Institut National de la Recherche Agronomique (INRA)	2018	Beef and dairy origin genotypes	Calorimetry/ NE	NE is quantified using the barley feed unit (FU), where 1 FU corresponds to 1760 kcal for 1 kg of fresh standard barley.
USA and Canada	National Academies of Sciences, Engineering, and Medicine (NASEM). Update on National Research Council (NRC) guidelines	2016	*Bos t. taurus*, *Bos t. indicus*, and crossbreds	Comparative slaughter/ NE	North American diets for feeding beef cattle are known for their high concentrate levels, distinguishing them from diets in other countries. The NASEM (2016) guidelines offer solutions from empirical to mechanistic approaches.
USA and Canada	Ruminant Nutrition System (RNS) Project	2018	*Bos t. taurus*, *Bos t. indicus*, and crossbreds	Comparative slaughter/ NE	The RNS (Ruminant Nutrition System) is an advancement of the Cornell Net Carbohydrate and Protein System, which was introduced in the 2000s. The RNS incorporates three levels of solutions (L0, L1, and L2), ranging from empirical to more mechanistic approaches.
Brazil	Universidade Federal de Viçosa (UFV) (BR-Corte)	2016	*Bos t. indicus* and crossbreds	Comparative slaughter/ NE	The predominant breed of Zebu cattle is Nellore, and energy equations have been developed for feedlot and pasture conditions. Calorimetry has been recently introduced as a method to estimate energy requirements.

For comparison purposes, energy systems can be grouped into two main categories. 1. metabolizable energy (ME) systems, which include AFRC and CSIRO, and 2. net energy (NE) systems, which include French (INRA), North American (NRC, NASEM, and RNS), and Brazilian (BR-Corte) systems. In UK and Australian systems, units for energy equations are in megajoules (MJ), whereas in North America and Brazil, calories are preferred. Feed units in the French system are usually converted to calorie equivalents. One calorie = 4.184 MJ.

**Table 4:** Equations used to calculate maintenance requirements (NE_m_, MJ/d) in growing animals *.

Systems	Equations
AFRC (1993)	$C(0.53{\left(LW/1.08\right)}^{0.67})0.0071LW$
CSIRO (2007)	$CKM\times 0.28LW^{0.75}e^{\left(-0.03A\right)}+0.1MEp\times K_{m}$
INRA (2018)	$0.289\;LW^{0.75}/0.423\;LW^{0.75}$
NASEM (2016)	$0.00293(20-Tp)+0.322\;SBW^{0.75}$
BR-Corte (2016)	$0.314\times {EBW}^{0.75}$

* For comparison purposes, energy coefficients are expressed in MJ (1 Mcal = 4.184 MJ); ME_m_ = NE_m_/k_m_. In AFRC, C = 1.0 for females and castrates and 1.15 for males. The factor 1.08 converts LW to fasted body weight; activity allowance: 0.0071 LW; LW = live weight. In CSIRO. A generalized equation without excluding energy expenditure at pasture and additional energy expenditure for low temperatures. C = as in the AFRC equation; K = 1.2 for *Bos taurus indicus*, 1.4 for *Bos taurus taurus*; M = is the fraction of the DE intake provided by milk. For convenience, where the proportion of milk in the diet is not known, M can be estimated from the following equation: M=1+(0.26−B×a), where B = 0.010 is a coefficient for suckled calves and a is week of life; a = age in years; MEp = the amount of dietary ME being used directly for production. In the INRA equation for growing and finishing beef, NE_m_(MJ/kg^0.75^) = 0.289 LW^0.75^ and 0.423 LW^0.75^ for pre-ruminant and ruminant animals, respectively; the NE_m_ increased from 88 kcal NE/kg^0.75^ to 101 kcal NE/kg^0.75^ in the updated version. The latest value was theoretically determined from feeding trials by regression techniques. In NASEM, Tp = ambient temperature; SBW = shrunk body weight; the equation can be further adjusted by multiplying it by a breed factor ranging from 0.9 (e.g., Brahman) to 1.2 (e.g., Holstein). In BR-Corte, the equation is valid for feedlot and pasture conditions; EBW = empty body weight.

**Table 5:** Metabolizable and NE requirements for maintenance for growing beef cattle from recent studies published around the world.

Ref.	Country	Technique	N	Type	Breed	LW (Kg)	ME_m_ (MJ/KG LW^0.75^)	NE_m_ (MJ/KG LW^0.75^)
**AFBI Studies (1990–2020)**								
[169]	UK	Calorimetry	20	Steers, heifers	Holstein	176	0.781	0.570
[168]	UK	Calorimetry	12	Steers	Angus × Friesian	416	0.620	-
[166]	UK	Calorimetry	75	Steers	Beef Cross	450–628	0.614	-
**International Studies (2009–2020)**								
[170]	Brazil	Comp. Slaughter	22	Heifers	Holstein × Gyr	98–172	0.545	0.352
[171]	Brazil	Calorimetry	15	Bulls	Holstein × Gyr	302	0.523	0.312
[172]	Brazil	Comp. Slaughter	39	Bulls	Holstein × Gyr	43–93	-	0.298
[173]	Brazil	Comp. slaughter	24	Bulls	Holstein × Gyr	182–388	-	0.313
[174]	Brazil	Calorimetry	5	Bulls	Nellore	219	0.691	0.418
	Brazil	Calorimetry	5	Bulls	Nellore	328	0.567	0.332
	Brazil	Calorimetry	5	Bulls	Nellore	394	0.512	0.331
	Brazil	Calorimetry	5	Bulls	Nellore	473	0.468	0.303
[175]	France	Feeding Studies	1855	Growing	Temperate and tropical	-	0.631	-
[176]	Brazil	Comp. Slaughter	752	Growing	Nellore, Nellore × *Bos taurus*	258–426	-	0.386
[177]	Brazil	Comp. Slaughter	44	Bulls	Holstein × Zebu	338	0.555	0.382
[178]	USA	Comp. Slaughter	127	Steers	Angus, Hereford, and cross	-	-	0.314
	Brazil	Comp. Slaughter	711	Bulls	*Bos indicus*	-	-	0.292
[179]	Brazil	Comp. Slaughter	46	Bulls	Nellore	138	0.603	0.325
[180]	Brazil	Comp. Slaughter	8	Steers	Nellore, High RFI	340–348	0.778	-
	Brazil	Comp. Slaughter	9	Steers	Nellore, Low RFI	334–441	0.637	-
[181]	Brazil	Comp. Slaughter	10	Bulls	Nellore × Holstein	199–317	0.607	0.352
**Summaries**								
AFBI studies (1990–2020)					0.672 ± 0.0947			
Literature (2009–2020)					0.593 ± 0.0846			
Cottrill et al. (1989–2009)					0.524 ± 0.0776			

Source: Cabezas-Garcia et al. [17] Refs. [68,96,97] are references for meta-analysis studies.

**Table 6:** Equations used to calculate energy requirements for LW gain (NE_g_) *.

Systems	Equations
AFRC (1993)	$C(4.1+0.0332LW-0.000009\;LW^{2})/(1-C_{2}0.1475\;LWG)$
CSIRO (2007)	$0.92[\left(6.7+R\right)+(20.3-R)/(1+e^{\left(-6\left(P-0.4\right)\right)})]$
INRA (2018)	22.9 ProtGain+39.3 LipGain
NASEM (2016)	$0.266EBW^{0.75}\times EBG^{1.097}$
BR-Corte (2016)	$0.052\times {EQEBW}^{0.75}\times {EBG}^{1.062}$

* For comparison purposes, energy coefficients are expressed in MJ (1 Mcal = 4.184 MJ); AFRC energy value of weight gain; C = 0.70 to 1.30 for different maturity (early, medium, and late) of different animals (bull, steer, and female); C_2_ = 1 when plane of nutrition, L > 1, and = 0 when L < 1 (L > 1 indicates that the animal is being fed above its maintenance requirements, and L < 1 indicates that the animal is being fed below its maintenance requirements); LW = live weight; LWG = live weight gain; CSIRO. Equation for immature animals (energy value of gain); 6.7 and 20.3 are coefficients expressing total energy in MJ/kg. R = adjustment for rate of gain or loss; P = live weight/standard reference weight; INRA. ProtGain and LipGain are protein and lipid deposition (kg/d); MEg = NE_g_/k_pf_; NASEM. Retained energy; EBW = empty body weight; EBG = empty body gain; BR-Corte. EQEBW = equivalent empty body weight. This is obtained by dividing the EBW by the weight at maturity of the respective sex/genetic group and multiplying by the reference weight; EBG = empty body gain.

**Table 7:** Equations used to calculate efficiencies of ME utilization for maintenance, growth, and lactation in the global energy system and calculated values at two diet metabolic ability values.

System	Equation	ME/GE *	
		0.50	0.65
AFRC (1993); CSIRO (2007)	$K_{m}\;=0.35\;ME/GE+0.503$	068	0.73
	$K_{g}\;=0.78\;ME/GE+0.006$	0.40	0.51
	$K_{l}\;=0.35\;ME/GE+0.42$	0.60	0.65
INRA (2018)	$K_{m}\;=0.287\;ME/GE+0.554$	0.70	0.74
	$K_{f}\;=0.78\;ME/GE+0.006$	0.40	0.51
	$K_{mf}\;={(K}_{m}\times K_{f}\times 1.5)/\;{(K}_{f}+0.5\times K_{m})\;$	-	-
	$K_{pf}=0.35+0.25\times {\left(1-EP\right)}^{2}$	-	-
	$K_{ls}=0.65+0.247\;(ME/GE-0.63)$	0.62	0.65
NASEM (2016)	$K_{m}\;=\left(1.37ME-0.138ME^{2}+0.0105ME^{3}-1.12\right)/ME$	0.61	0.67
	$K_{g}\;=\left(1.42ME-0.174ME^{2}+0.0122ME^{3}-1.65\right)/ME$	0.35	0.45
BR-Corte (2016)	$K_{m}\;=[\left(0.513+0.173\times K_{g}+\beta_{2}\times EBG\right)\times \theta ]$	-	-
	$K_{g}\;=0.327/(0.539-REp)$	-	-

* ME/GE is metabolic ability. CSIRO. Although the Australian system largely adopted the principles and equations used in the AFRC, those equations were converted to M/D (MJ of ME per kg of feed DM). There is a specific equation for k_g_ in grazing conditions: k_g_ = 0.035 M/D (1 + 0.33 Le) (1.0 + 0.12(λ sin (0.0172 T)/40)); where Le = the proportion of legume in the forage, T = the day of the year from 1 January, h = the latitude (◦) of the site; adverse in the south. Otherwise, k_g_ in the Table above (converted to M/D equivalents) is recommended for concentrate and grass silage-based diets; INRA. The ME units = Mcal/kg of DM; EP = protein proportion in LW gain. EP = 5.48 ProtGain/(5.48 ProtGain + 9.39 LipGain); For slow-growing cattle (LW gain ≤ 1 kg/d, a metabolizable coefficient is calculated instead as qprimma = 0.62−0.262 × exp(−3.175 × LW gain), with LW gain in kg/d; k_f_ = fattening; k_mf_ = combined efficiency of ME for maintenance, growth, and meat deposition for fast-growing animals; k_pf_ = protein and fat deposition (known body gain composition); k_ls_ = milk yield + maintenance for lactating animals/maintenance and gain for slow-growing cattle. Both k_g_ and k_l_ as such are used as such in the up-to-date version of the French system. NASEM, the same equations for k_m_ and k_g_ as in the NRC. Values for energy efficiencies (k_s_) are not based on diet metabolizable (ME/GE); BR-Corte, the efficiency for maintenance, includes k_g_; EBG = empty body gain (kg/d), β_2_ = 0.100 for Zebu, 0.073 for beef crossbred, and 0.010 for dairy crossbred, and θ = fit factor for the rearing system that takes the value of 1 for animals reared on feedlot and 0.92 for pasture-reared animals. There is no explicit mention of k_l_ calculations. Rather than providing information to estimate energetic efficiencies, the NASEM included equations to calculate dietary NE concentrations for maintenance and LW gain. For comparison purposes, the k_m_ and k_g_ values in NASEM are estimated by dividing NE data by ME values as proposed by Cottrill et al.; see Table 5, when assuming metabolizable coefficients of 0.50 and 0.65. Conversely, in the Brazilian system, the principle for calculating both k_m_ and k_g_ values is not based on the ME/GE ratio. Instead, both animal-related and production-system factors are considered. The authors did not obtain accurate k_g_ predictions based on ME concentration in the diet.

**Table 8:** Regression of logarithm of heat production on ME intake to describe energy utilization by Nellore and *B. taurus* × Nellore crosses steers ^1^.

GG ^2^	Intercept ^2^	Slope (×1.000)	N	r ^2^	RMSE	NE_m_	ME_m_	k_m_(CI)	k_g_(CI)
AN	1.96±0.025	1.374±0.086	16	0.94	0.015	90.76	142.44	63.7 (56.3,69.3)	28.4% (14.6, 42.2)
CN	1.92±0.014	1.541±0.052	16	0.98	0.009	82.28	130.98	62.8 (60.7, 66.2)	22.1% (8.4, 35.9)
NL	1.93±0.029	1.495±0.107	16	0.93	0.016	85.53	137.12	62.4 (62.8, 69.1)	24.6% (10.4, 38.8)
SN	1.95±0.044	1.401±0.168	14	0.85	0.024	88.80	139.11	63.8 (46.6, 73.3)	29.5% (29.5, 29.5)
All	1.94±0.013	1.450±0.049	62	0.93	0.016	86.86	137.53	63.2 (59.3, 66.5)	26.0% (23.3, 28.6)

^1^ Values are mean ± SE. RMSE, the root of the mean square error; NE_m_, net energy required for maintenance (kcal/kg^0.75^ of EBW per d), calculated as the antilog of the intercept; ME_m_, metabolizable energy required for maintenance (kcal/kg^0.75^ of EBW per d) calculated by iteration assuming heat produced is equal to ME intake at maintenance; km, efficiency of energy utilization for maintenance (calculated as NE_m_/ME_m_); k_g_, efficiency of energy utilization for growth, which was calculated as the slope of the regression of RE (kcal/kg^0.75^ EBW) on ME intake (kcal/kg^0.75^ EBW). CI, the confidence interval for k_m_ and k_g_, was computed by adding or subtracting one SE of the intercept and slopes for each genetic group (GG) and calculating the NE_m_, ME_m_, k_m_, and k_g_ as described earlier. ^2^ NL, Nellore; AN, one-half Angus + one-half Nellore; CN, one-half Canchim (five-eighths Charolais + three-eighths Zebu) + one-half Nellore; SN, one-half Simmental + one-half Nellore. The limit-fed treatment received 70% of the daily feed of the ad libitum-fed treatment of the same genetic group.

## Data Availability

Not applicable.
